# ADAR-deficiency perturbs the global splicing landscape in mouse tissues

**DOI:** 10.1101/gr.256933.119

**Published:** 2020-08

**Authors:** Utkarsh Kapoor, Konstantin Licht, Fabian Amman, Tobias Jakobi, David Martin, Christoph Dieterich, Michael F. Jantsch

**Affiliations:** 1 Center of Anatomy and Cell Biology, Department of Cell and Developmental Biology, Medical University of Vienna, A-1090 Vienna, Austria;; 2 Institute of Theoretical Biochemistry, University of Vienna, A-1090 Vienna, Austria;; 3 Department of Internal Medicine III and Klaus Tschira Institute for Computational Cardiology, Section of Bioinformatics and Systems Cardiology, University Hospital, D-96120 Heidelberg, Germany

## Abstract

Adenosine-to-inosine RNA editing and pre-mRNA splicing largely occur cotranscriptionally and influence each other. Here, we use mice deficient in either one of the two editing enzymes ADAR (ADAR1) or ADARB1 (ADAR2) to determine the transcriptome-wide impact of RNA editing on splicing across different tissues. We find that ADAR has a 100× higher impact on splicing than ADARB1, although both enzymes target a similar number of substrates with a large common overlap. Consistently, differentially spliced regions frequently harbor ADAR editing sites. Moreover, catalytically dead ADAR also impacts splicing, demonstrating that RNA binding of ADAR affects splicing. In contrast, ADARB1 editing sites are found enriched 5′ of differentially spliced regions. Several of these ADARB1-mediated editing events change splice consensus sequences, therefore strongly influencing splicing of some mRNAs. A significant overlap between differentially edited and differentially spliced sites suggests evolutionary selection toward splicing being regulated by editing in a tissue-specific manner.

RNA modifications affect composition, stability, structure, and function of messenger RNAs (Licht and Jantsch 2016). In metazoans, adenosine-to-inosine (A-to-I) RNA editing is the most abundant type of RNA editing and is mediated by the adenosine deaminase acting on RNA (ADAR) family of enzymes (Nishikura 2016; Eisenberg and Levanon 2018). During A-to-I editing, an inosine is generated by hydrolytic deamination of adenosines. Inosines are primarily read as guanosines by cellular machines and occasionally as adenosines or uracils (Basilio et al. 1962; Licht et al. 2019a). In mammals, two types of active ADARs, ADAR (ADAR1) and ADARB1 (ADAR2), are found that modify different but partially overlapping substrate sites (Eggington et al. 2011).

ADARs bind double-stranded RNA (dsRNA) which can be formed between different regions of an RNA. In mRNAs, this can involve exon-intron, exon-exon, or intron-intron base-pairing. We recently showed that, in the mouse, most editing-competent structures are formed within introns (intron-intron base-pairing), followed by structures formed within UTRs (Licht et al. 2019b). The definition of some editing sites by base-pairing between exonic and intronic sequences has led to the notion that A-to-I editing must occur cotranscriptionally, or before intron removal (O'Connell 1997). Splicing itself occurs mostly cotranscriptionally (Merkhofer et al. 2014). Consistently, both pre-mRNA splicing and RNA editing are coordinated (Bratt and Ohman 2003). Moreover, both processes contribute to proteomic diversity (Wang and Burge 2008).

Splicing efficiency can control editing levels. Both mini-gene reporter assays as well as analyses of endogenous targets demonstrated that exon-intron-dependent editing sites are strongly affected by the efficiency of splicing (Licht et al. 2016). Conversely, A-to-I RNA editing may affect splicing by creating or disrupting splice sites or branch points (Rueter et al. 1999). Similarly, ADARs may alter binding sites for splicing factors and compete with splicing factors for binding and/or access to the same RNA. Several studies have shown an impact of RNA editing on splicing for selected substrates. For instance, inhibition of editing of the glutamate receptor subunit *Gria2* impairs splicing of intron 11 and affects alternative splicing at intron 13/14 (Higuchi et al. 2000; Schoft et al. 2007; Penn et al. 2013). Similarly, global studies performed in human tissue culture cells, flies, and mouse brains lacking ADARB1 have provided insights into the impact of editing on splicing (Solomon et al. 2013; St Laurent et al. 2013; Mazloomian and Meyer 2015; Dillman et al. 2016; Hsiao et al. 2018). However, a transcriptome-wide splicing analysis comparing different tissues within a mouse genetic deletion model of *Adar* remains elusive.

In mice, both ADARs are essential but can be rescued to different extents. *Adar*-null mice are embryonic lethal and die at stage E11.5 and show defects in erythropoiesis, elevated interferon signaling, and widespread apoptosis (Hartner et al. 2004, 2009; Wang et al. 2004; Liddicoat et al. 2015). It has been shown that a deletion in *Adar* can be rescued by a concomitant deletion of the gene encoding the cytoplasmic RNA sensor IFIH1 (also known as MDA5) or the gene encoding its downstream signaling mediator MAVS. The extent to which *Adar* deficiency can be rescued depends strongly on the *Adar* allele used and ranges from complete viability, over reduced growth, to postnatal death (Mannion et al. 2014; Liddicoat et al. 2015; Pestal et al. 2015; Bajad et al. 2020). Similarly, *Adarb1*-null mice die within a few weeks after birth accompanied by seizures and epilepsy. *Adarb1* deficiency can be rescued by a pre-edited version of the AMPA glutamate receptor subunit 2 (*Gria2*) (Higuchi et al. 2000). These mice have been extensively studied and appear phenotypically normal under standard laboratory conditions (Higuchi et al. 2000). The *Adar* deletion mice rescued by a concomitant *Mavs* deletion exhibit a minute phenotype (Bajad et al. 2020).

In this study, for the first time, we use *Adar*-deficient, rescued mice to characterize the ADAR-mediated impact on the transcriptome-wide splicing landscape in different mouse tissues and compare their splicing landscape with that of *Adarb1*-deficient rescued mice (Higuchi et al. 2000).

## Results

### RNA-seq and global splicing analysis

To determine the impact of ADAR on splicing, we interbred *Mavs*^−/−^ mice with *Adar*^+/−^ (*Adar*^Δ7-9^) mice to generate *Adar*^*+/−*^ ; *Mavs*^*−/−*^ mice (Bajad et al. 2020). Offspring of these mice with genotype *Adar*^*+/+*^ ; *Mavs*^*−/−*^ (*Adar* WT) and *Adar*^*−/−*^ ; *Mavs*^*−/−*^ (*Adar* KO) were collected at P14. In the *Adar*^*−/−*^ mice used here, a truncated, editing-deficient ADAR protein is expressed (Bajad et al. 2020). To assess the impact of ADARB1 on splicing, we crossed heterozygous *Adarb1*^+/−^ ; *Gria2*^R/R^ mice and collected *Adarb1*^+/+^ ; *Gria2*^R/R^ (*Adarb1* WT) and *Adarb1*^−/−^ ; *Gria2*^R/R^ (*Adarb1* KO) pups at P14.

**Figure 1.**
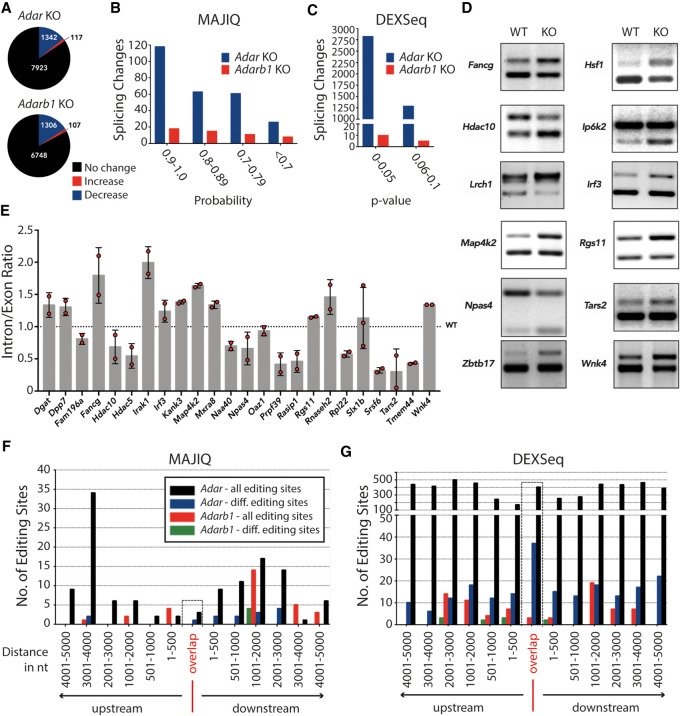
*ADAR* or *ADARB1* deficiency causes transcriptome-wide changes in splicing patterns. (*A*) Pie charts depicting differential editing analysis in *Adar*- or *Adarb1*-deficient cortex. Editing levels remain steady (black), increase (red), or decrease (blue). (*B*) Histogram showing local splicing variations (LSVs) identified by MAJIQ tool in ADAR (*Adar*-) or *Adarb1*-deficient cortex binned by MAJIQ probability score. (*C*) Histogram showing differential exon/intron usage events identified by DEXSeq in *Adar*- or *Adarb1*-deficient cortex binned by DEXSeq adjusted *P*-value. (*D*) RT-PCR validation of LSVs predicted by MAJIQ in *Adar*-deficient cortex resolved by agarose gel electrophoresis. (*E*) qPCR validation of LSVs predicted by MAJIQ in *Adar*-deficient cortex. Data shown are the mean inclusion to exclusion ratio in *Adar* KO (±SD). (*F*) Histogram showing ADAR and ADARB1 differential editing sites found in the indicated distances of ADAR- and ADARB1-dependent LSV events identified by MAJIQ in the cortex; editing sites are binned (±5 kb) by significant chromosomal coordinates. Editing sites that lie exactly on/within the differentially spliced regions have been highlighted and binned as “Overlap.” Only those editing sites that were found in the ±5-kb window have been plotted. (*G*) Histogram showing ADAR and ADARB1 differential editing sites identified in the indicated distances of ADAR- and ADARB1-dependent differential exon/intron usage identified in the cortex; editing sites are binned (±5 kb) by significant chromosomal coordinates. Editing sites that lie exactly on/within the differentially spliced regions have been highlighted and binned as “Overlap.” Only those editing sites that were found in the ±5-kb window have been plotted.

Since A-to-I RNA editing events are enriched in tissues of neuronal origin, we sequenced poly(A)-selected RNA of cortices isolated from *Adar* WT, *Adar* KO, *Adarb1* WT, and *Adarb1* KO mice in biological triplicates in 125-bp paired-end mode on an Illumina HiSeq 2500 (Supplemental Fig. S1A,B). Editing sites were detected in these RNA-seq data sets using a machine learning algorithm RDDpred (Kim et al. 2016). Editing levels for each site were calculated by dividing the number of edited reads by the total number of reads spanning the editing site. Following removal of known SNPs and stringent filtering, we compiled a list of differentially edited sites where we observed a significant change (Welch's *t*-test; *P* ≤ 0.1) in RNA editing levels between WT and KO cortex samples. We detected 9382 editing sites in *Adar* WT, out of which 1459 were found to be differentially edited in the *Adar* KO cortex. Similarly, we detected 8161 editing sites in *Adarb1* WT, out of which 1413 were found to be differentially edited in the *Adarb1* KO cortex. As expected, most differentially edited sites showed reduced editing upon *Adar* or *Adarb1* deletion. In contrast, editing levels were increased for a small number of editing sites upon depletion of one of the two ADARs (Fig. 1A). In the *Adar*^−/−^ cortex, 117 sites showed an increase in editing between 1% and 83%. Similarly, in *Adarb1*^−/−^ cortex, 107 sites showed an increase in editing between 1% and 54% (Supplemental Fig. S2A,B; Supplemental Dataset 1). This observation suggests competition between ADAR and ADARB1 for access to the same editing site that hints toward a regulatory mechanism to keep editing levels in check.

Next, we profiled global splicing changes using the Modeling Alternative Junction Inclusion Quantification (MAJIQ) tool (Vaquero-Garcia et al. 2016). MAJIQ defines differential splicing events as local splicing variations (LSVs). An LSV encompasses typical forms of alternative splicing like exon skipping, mutually exclusive exons, intron retention, and alternative 5′ or 3′ splice sites but also includes nonstandard events resulting in complex splicing patterns (Supplemental Fig. S3; Vaquero-Garcia et al. 2016). We obtained 269 LSV events in 141 genes from RNA-seq data analysis of *Adar* KO cortex and 52 LSVs in 35 genes in *Adarb1* KO cortex (Fig. 1B; Supplemental Dataset 2). MAJIQ generates a probability score P which estimates whether the difference of splice junction usage (delta Ψ) between experimental conditions is greater than or equal to 20% (P [delta Ψ] ≥ 0.2]. Higher probability suggests high confidence in the predicted differential splicing event. For RT-PCR and qRT-PCR validations, LSVs that had a probability score ≥ 0.6 were picked (Fig. 1D,E). Out of 24 tested targets with probability values ranging between 0.62 and 0.99, 19 were positively validated to have a significant change in inclusion to exclusion ratio in at least two replicates indicating a false discovery rate of ∼20%.

Several substrates previously shown to be differentially spliced upon loss of editing (e.g., *Gria2, Htr2c*) were not identified in the MAJIQ analysis (Flomen et al. 2004; Licht et al. 2016). MAJIQ computes relative inclusion of isoforms but does not quantify expression levels of isoforms. Thus, we decided to complement our analysis using DEXSeq (Anders et al. 2012) which evaluates differential exon usage between samples from RNA-seq data. We adjusted the DEXSeq analysis in order to allow quantification of differential intron usage, thereby allowing analysis of intron retention events. We found 4113 events in 3010 genes in *Adar* KO and only 15 events in nine genes in *Adarb1* KO that were significantly different (adjusted *P*-value ≤ 0.1) (Fig. 1C; Supplemental Dataset 3). Not only did DEXSeq predict a much higher number of differential exon/intron usage events in *Adar* KO cortex, but also transcripts with splicing events known to be affected by editing were detected. The overlap between events identified using MAJIQ and DEXSeq was only moderate (51 targets in the *Adar* KO cortex), suggesting that both algorithms complement each other (Supplemental Fig. S4). For qRT-PCR validations of differential exon/intron usage events predicted by DEXSeq, targets with an adjusted *P*-value ≤ 0.1 were chosen. Out of 29 tested targets with adjusted *P*-values ranging between 0.0 and 0.09, 22 were positively validated to have a significant change in inclusion to exclusion ratio in at least two out of three replicates indicating a false discovery rate of ∼25% (Supplemental Figs. S5, S6). Among those, the transcript encoding *Dicer* was differentially spliced in *Adar* KO cortex (Supplemental Fig. S5A). Also, *Adat2* had a significant differential exon usage event in two out of three replicates in the *Adar* KO cortex. Further, *Adarb1* transcripts displayed a significant differential intron usage in three out of three replicates in the *Adar* KO cortex, likely leading to reduced ADARB1 protein expression, as the retained intron would reduce mRNA expression (Braunschweig et al. 2014; Wong et al. 2016). In line with this, we observed reduced editing levels for several ADARB1-dependent editing sites in the *Adar* knockout cortex (Supplemental Fig. S7).

To identify editing sites that are associated with predicted splicing changes, we looked for enrichment of differential *Adar* or *Adarb1* editing sites overlapping with or within a window (±5 kb) of the significantly differentially spliced regions detected in either *Adar* or *Adarb1* KO cortex. In the MAJIQ data set, only one differential editing site overlapping a MAJIQ event was detected in *9930104L06Rik* in the *Adar* KO cortex, whereas no editing sites were detected that overlapped a MAJIQ event in *Adarb1* KO cortex (Fig. 1F “overlap”). Out of a total of 14 differential editing sites that were found within ±5 kb of differentially spliced MAJIQ coordinates in the *Adar* KO cortex data set, two editing sites were found upstream, one overlapped, and 11 were downstream from the event. On the contrary, four differential editing sites were detected only downstream from differentially spliced MAJIQ coordinates in the *Adarb1* KO cortex data set (Fig. 1F). A similar analysis was performed using the *Adar*/*Adarb1* KO DEXSeq data sets (Fig. 1G). Out of a total of 207 differential editing sites that were found within ±5 kb of differentially spliced DEXSeq coordinates in the *Adar* KO cortex data set, 72 editing sites were found upstream, 37 overlapped, and 98 were found downstream from the event. On the contrary, in the *Adarb1* KO cortex data set, 10 differential editing sites were detected either upstream of or downstream from the differentially spliced DEXSeq coordinates, whereas none overlapped (Fig. 1G). These include editing sites in *Flna and Flnb*.

In the *Adar* KO cortex, we found 23 genes that harbored 37 differential editing sites within the coordinates of a differential exon/intron usage event (Fig. 1G, “overlap”; Supplemental Table S1). These genes include *Alkbh2, Mcat,* or *Pin1* harboring intronic editing sites and *Mrps17* which contains five differentially edited sites in the 3′ UTR. From this list, at random we picked four genes: *Dusp11*, *Eloc*, *Pin1,* all harboring intronic editing sites, and *Ezh1*, having a differentially edited site in the 3′ UTR and validated them by qPCR. In three candidates, *Dusp11*, *Ezh1*, and *Pin1*, we observed the predicted trend in at least two out of three replicates in *Adar* KO cortex (Supplemental Fig. S5B). PIN1 [protein (peptidyl-prolyl cis/trans isomerase) NIMA-interacting 1] is known to regulate *Gria2* Q/R site RNA editing by binding to ADARB1 in a phosphorylation-dependent manner (Marcucci et al. 2011). In the *Adar* KO cortex, we found *Pin1* to be differentially edited in intron 1, where we observed editing levels at this site to drop from 11% to 0%. We validated the same intron 1 to have a higher intron retention ratio in *Adar* KO cortex, leading to a premature termination codon and thus likely reducing PIN1 levels (Braunschweig et al. 2014; Wong et al. 2016). This suggests that ADAR could also regulate ADARB1-dependent RNA editing via splicing of *Pin1.* Consistently, we observed a 50% drop in editing levels for several ADARB1-dependent editing sites upon *Adar* deletion, supporting the idea that ADAR may lead to reduced ADARB1 activity by reducing *Pin1* expression (Supplemental Fig. S7; see above).

We also validated differential intron 15 usage in *Ezh1*, a component of the Polycomb Repressive Complex 2 (PRC2) (Margueron and Reinberg 2011). This region has four differential editing sites showing the highest change in editing levels from 46% to 0% in the *Adar* KO cortex. Additionally, *Dusp11*, which encodes an RNA-binding protein, was validated to have differential intron 3 usage with an editing site showing a concomitant decrease in editing levels from 25% to 0% in *Adar* KO cortex (Supplemental Fig. S5B). Out of 23 genes that had differential editing sites overlapping a DEXSeq event, two genes, *Pnpla6* and *Rbbp4*, harbored intronic sites that showed an increase in editing levels from 1% to 25% and 7% to 26%, respectively, in the *Adar* KO cortex (Supplemental Table S1).

To obtain insights into genes that were detected to be differentially spliced in the MAJIQ and DEXSeq data sets, the Enrichr tool (Kuleshov et al. 2016) was used to determine enrichment of Gene Ontology (GO) terms in the DEXSeq and MAJIQ data sets. In both, we found significant hits on ontology terms linked with splicing, such as regulation of splicing via spliceosome, RNA processing, and gene expression (Supplemental Tables S2, S3). This suggests that ADARs can have a broad impact on pre-mRNA splicing by regulating splicing factors that are involved or associated with splicing.

### The impact of ADAR on splicing is tissue-specific

Since ADAR seemingly has a larger impact on splicing than ADARB1 in the cortex, we asked if ADAR had any tissue-specific effect on splicing. To answer this, we used RNA-seq data from *Adar* WT and *Adar* KO bone marrow and liver and profiled the global editing as well as splicing landscape in these tissues (Bajad et al. 2020). These RNA-seq libraries were prepared from ribo-minus RNA samples and sequenced in 125-bp paired-end mode using the Illumina HiSeq 2500 (Supplemental Fig. S1A,B).

**Figure 2.**
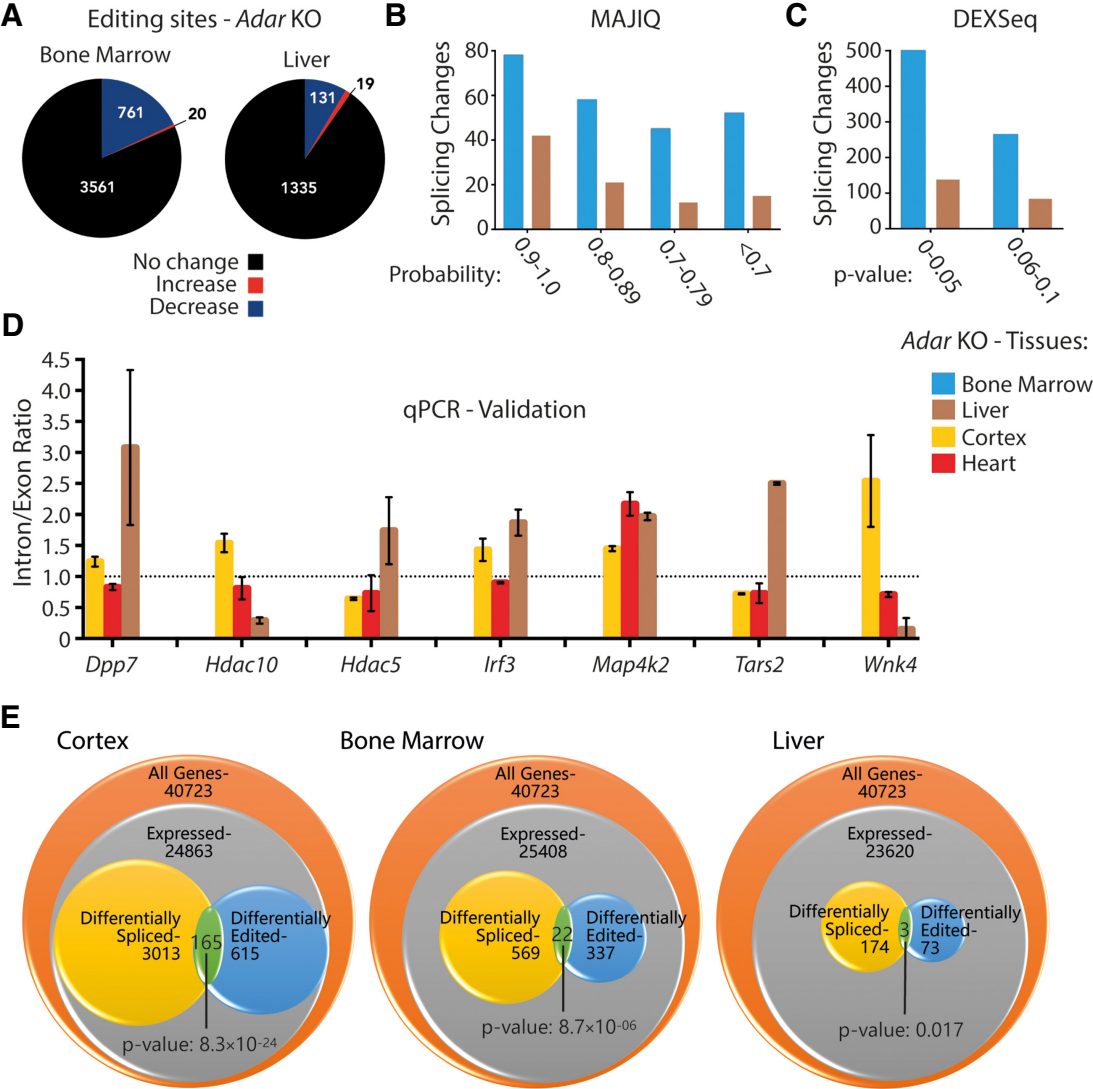
ADAR-dependent splicing changes are tissue-specific. (*A*) Pie charts depicting the differential editing analysis in *Adar*-deficient bone marrow and liver. Editing levels remain steady (black), increase (red), or decrease (blue). (*B*) Histogram showing local splicing variations identified by MAJIQ in *Adar*-deficient bone marrow (blue) and liver (brown) binned by the MAJIQ probability score. (*C*) Histogram showing differential exon/intron usage events identified by DEXSeq in *Adar*-deficient bone marrow (blue) and liver (brown) binned by the DEXSeq adjusted *P*-value. (*D*) Histogram showing inclusion/exclusion ratios (liver: brown, cortex: yellow, heart: red) validated by qPCR of MAJIQ events (*n* = 2 or 3). Data shown are the mean inclusion to exclusion ratio in *Adar* KO (±SD). (*E*) *GeneOverlap* analysis of the number of genes expressed per tissue, those that show differential editing, and those that show differential splicing. The overlap between the latter two categories is higher than stochastically expected, suggesting that editing and splicing are mechanistically linked.

In order to look for editing sites that are differentially edited across *Adar*-deficient tissues, we first performed editing site detection in *Adar* WT and *Adar* KO bone marrow and liver RNA-seq data sets using RDDpred (Kim et al. 2016). In all three tissues (cortex, bone marrow, liver) only 5%–10% of editing sites were annotated as exonic whereas the majority of editing sites located to intronic regions (Supplemental Fig. S8). The different modes of library preparation (cortex: poly(A)-selected vs. liver and bone marrow: ribo-minus) possibly reflect the higher ratio of intronic versus exonic editing sites in bone marrow and liver. Next, we compiled a list of differentially edited sites where we observed a significant change (Welch's *t*-test; *P* ≤ 0.1) in RNA editing levels between WT and KO samples. We found 805 (out of 4366) and 150 (out of 1485) editing sites to be differentially edited (*P* ≤ 0.1) in *Adar* KO bone marrow and liver, respectively (Fig. 2A; Supplemental Fig. S2C,D; Supplemental Dataset 1). This is consistent with the notion that A-to-I editing generally reaches the highest complexity in brain tissues (Heraud-Farlow et al. 2017; Tan et al. 2017) but is also in good agreement with the different read coverage in those tissues: ∼170 million in bone marrow, ∼131 million in liver, ∼465 million in cortex.

Next, the global splicing landscape was profiled in bone marrow and liver data sets using both MAJIQ and DEXSeq. MAJIQ identified 233 local splicing variations in 94 genes in *Adar* KO bone marrow and 90 LSVs in 49 genes in *Adar* KO liver (Fig. 2B). Similarly, DEXSeq identified 764 differential exon/intron usage events in 569 genes in *Adar* KO bone marrow and 222 events in 174 genes in *Adar* KO liver (Fig. 2C). As mentioned, the lower number of events detected in these tissues is likely due to less depth in RNA-seq data (number of reads) but could also be attributed to the lesser splicing activity compared to tissues of neuronal origin (Raj and Blencowe 2015).

Next, the output of significantly altered splicing events from *Adar* KO cortex, *Adar* KO bone marrow, and *Adar* KO liver was intersected. Seven genes were commonly called differentially spliced in all tissues. However, neither MAJIQ nor DEXSeq (Supplemental Fig. S9A) identified the same splicing event within those genes to be similarly affected across all tissues. Although MAJIQ did not find any LSVs that were common across all tissues, we tested if the MAJIQ-predicted targets in the *Adar* KO cortex could have a tissue-specific outcome. For this, we chose LSVs that were already verified in the *Adar* KO cortex and validated them in other *Adar* KO tissues such as heart and liver by qPCR. Indeed, we found seven out of seven tested targets to behave in a tissue-specific manner (Fig. 2D). For instance, dipeptidylpeptidase 7 (*Dpp7*) and interferon regulatory factor 3 (*Irf3*) showed similar trends leading to higher inclusion in *Adar* KO cortex and liver but less inclusion in *Adar* KO heart. Histone deacetylase 5 (*Hdac5*) showed significantly altered splicing only in the *Adar* mutant cortex, whereas *Hdac10* showed significantly altered patterns in both mutant cortex and liver, albeit with opposite trends. Similarly, *Tars2* and *Wnk4* showed different, yet significant trends in all three tested tissues Only *Map4k2* showed significant and the same trends of higher inclusion across all tissues (Fig. 2D).

From the intersection of DEXSeq outputs, six events were found to be common between cortex and liver, nine between liver and bone marrow, and 18 between bone marrow and cortex (Supplemental Fig. S9A; Supplemental Table S4). However, the relative trend toward inclusion or exclusion differed strongly. Although eight out of nine events shared between bone marrow and liver showed the same trend for specific regions, this was true for only three out of six DEXSeq regions when comparing cortex and liver and 12 out of 18 regions for bone marrow versus cortex (Supplemental Table S4). From those significant events, we took four candidates at random and successfully validated them by qPCR. Candidates were validated in all three tissues even if they were only found common between any two tissues. DEXSeq generates adjusted *P*-values and the fragment IDs of these candidates are listed (Supplemental Fig. S9B). From qPCR analysis (Supplemental Fig. S9C), we find that mitochondrial ATP synthase (*Atp5b*) (Fragment: E010) and epidermal growth factor receptor (*Egfr*) (Fragment: E046) show similar trends leading to higher inclusion in the *Adar* KO in cortex and liver, while showing no change in the KO bone marrow. *Fgd1* (also known as faciogenital dysplasia) (Fragment: E020) shows different, yet significant trends in all three *Adar*-deficient tissues, that is, lesser inclusion in cortex and bone marrow whereas much higher inclusion is observed in the liver. Similarly, tetratricopeptide repeat domain protein 19 (*Ttc19*) (Fragment: E018) shows less inclusion in bone marrow and higher inclusion in liver, while showing no change in the cortex upon *Adar* deletion. It should be noted that, although all four targets showed significant changes by qPCR, they did not match the DEXSeq predicted trend in all tissues (Supplemental Fig. S9C). *Atp5b* showed the expected trend in cortex but not in bone marrow, whereas *Egfr* met the expected trend in both cortex and liver. Similarly, *Fgd1* and *Ttc19* showed the expected trend in liver but not in bone marrow. We currently cannot explain this observation. We also checked the gene expression profile of these targets and found no significant change in their mean transcripts per million (TPM) values between *Adar* WT and *Adar* KO. Although TPM values are different between tissues, they do not change between WT and KO in the same tissue (Supplemental Fig. S9D; Supplemental Dataset 4). Overall, this led us to conclude that ADAR has a different impact on splicing in different tissues, with no particular trend toward inclusion or exclusion.

Given that we found a number of genes that responded to loss of *Adar* by showing changes in editing or splicing levels, we asked if this number of altered splicing events hinted toward a regulatory impact of editing on splicing. If this was the case, one would expect the overlap between editing events and alternative splicing events to be significantly larger than a stochastic model would predict. We therefore used *GeneOverlap*, a Bioconductor R package that uses the principle of Fisher's exact test to evaluate the statistical significance of overlap between any two gene lists normalized to the genomic/transcriptomic background. Since DEXSeq was able to predict a higher number of differential exon/intron usage events in all tested *Adar*-deficient tissues, we used only DEXSeq genes for this analysis. When testing for an enriched overlap between the list of genes that have a significant differential editing site and the list of genes that have a significant DEXSeq event, we observed a significant enrichment in all tested *Adar*-deficient DEXSeq data sets. The effect was most pronounced in the cortex where altered editing and splice pattern co-occurred in 165 genes (*P*-value 8.3 × 10^−24^), followed by bone marrow with 22 overlapping genes (*P*-value 8.7 × 10^−10^), and liver with three overlapping genes (*P*-value 0.017). This shows that editing and splicing are significantly linked across tissues (Fig. 2E).

### A-to-I RNA editing in the immediate vicinity (<50 nt) of exon-intron boundaries influences splicing efficiency in a position-independent manner

**Figure 3.**
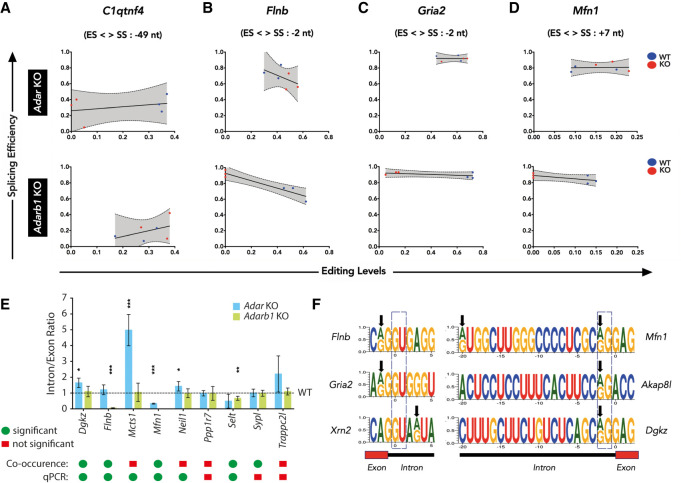
Nearby editing and splicing events are frequently linked. (*A*–*D*) Linear regression of co-occurrence analysis comparing editing levels and splicing levels of *C1qtnf4*, *Flnb*, *Gria2*, and *Mfn1* in *Adar* KO and *Adarb1* KO cortex samples. WT samples are shown in blue and KO samples are shown in red. The editing site position (ES) relative (upstream: +, or downstream: −) to the splice site position (SS) is given. (*E*) Histogram showing qPCR validation of targets identified by co-occurrence analysis in *Adar* KO and *Adarb1* KO cortex. Data shown are mean inclusion to exclusion ratio (±SD). Statistical test performed with Student's *t*-test; (*) *P* < 0.05, (**) *P* < 0.01, (***) *P* < 0.001. A comparison of the significance found in the co-occurrence analysis of NGS data by Fisher's exact test and the output of the qPCR experiments as calculated by Student's *t*-test. Green dots = significant; red squares = not significant. (*F*) Schematic representing examples of positions of editing sites in the 5′ splice sites of *Flnb, Gria2*, and *Xrn2* as well as in the 3′ splice sites of *Mfn1*, *Akap8l*, and *Dgkz*. Black arrows show the location of editing sites. A dotted box highlights the canonical 5′ GU and 3′ AG splice sites.

The majority of differentially spliced events called and validated in the MAJIQ and DEXSeq data sets do not contain differentially edited sites within the affected region. It therefore seems likely that RNA editing affects these splicing events only indirectly. To identify differentially spliced targets that are directly affected by editing, a co-occurrence analysis was performed. In short, individual reads spanning a potential splice site and nearby editing site (±50 nt) were scanned for the co-occurrence of editing and splicing events. For this analysis, RNA-seq data generated from *Adar* and *Adarb1* WT/KO cortex were used. Forty-six editing site-splice site combinations were identified in 32 genes, which included known editing targets like *Adarb1, Grik2*, or *Neil1*, where a particular splice pattern correlated with the presence or absence of an editing event (Supplemental Dataset 5). Out of 46 editing-splice site combinations, 12 were significant (Fisher's exact test, multiple testing corrected *P*-value ≤ 0.1). Comparison of editing levels and splicing levels by linear regression shows that A-to-I RNA editing influences splicing efficiency irrespective of the position of the editing site with respect to the exon/intron boundary (Fig. 3A–D). This observation is limited to the editing sites in the chosen distance (±50 nt).

For instance, *C1qtnf4* is an ADAR target, as editing levels in *Adar* KO are consistently reduced whereas editing levels in the *Adarb1* KO vary (Fig. 3A). Accordingly, splicing levels were consistent in at least two out of three *Adar* KO cortex samples but not in the *Adarb1* KO cortex. Furthermore, *Flnb, Gria2*, and *Mfn1* are ADARB1 targets exhibiting consistent loss in editing levels with a concomitant increase in splicing efficiency in *Adarb1* KO while displaying no particular trend in *Adar* KO (Fig. 3B–D), indicating that editing in these targets reduces splicing efficiency. The consistency of editing-splicing levels can be appreciated by the clear separation and clustering of WT and KO data points in the respective genotypes. The editing site in *Mfn1* is located at the 5′ end of the intron, which may affect U1 base-pairing (Fig. 3F; Supplemental Fig. S10). In contrast, the editing site in *C1qtnf4* is located within the intron and may lead to refolding of the intron (Supplemental Fig. S10). Editing at the *Gria2* R/G-site or *Flna* and *Flnb* sites is located close to the 5′ splice site at position −2 (Higuchi et al. 1993; Czermak et al. 2018).

Combination of reads that span editing site and splice site were factored in when determining the statistical significance of co-occurrence using Fisher's exact test (see Methods). To estimate the reliability of these results, we randomly chose nine editing site-splice site combinations and validated them by qPCR. We found that six out of nine targets were in agreement with the prediction from co-occurrence analysis showing a significant change in inclusion to exclusion ratio in either *Adar* or *Adarb1* knockout cortex (Fig. 3E). The two targets *Mcts1* and *Neil1* predicted to have insignificant co-occurrence were found to be significant by qPCR (Fig. 3E). Overall, this led us to conclude that a co-occurrence analysis is a reliable strategy to identify splicing events that are affected by A-to-I RNA editing events near the exon-intron boundary.

Next, we examined if editing could have an impact on the strength of the splice sites. For this analysis, we used the MaxEntScan program that uses a maximum entropy principle to model sequence motifs near exon-intron boundaries (Yeo and Burge 2004). Specifically, it models nine bases at the 5′ splice site (−3 nt in exon and +6 nt in intron) and 23 bases at the 3′ splice site (−20 nt in intron and +3 nt in exon). Here, all currently known editing sites in the mouse from RADAR (Ramaswami and Li 2014) and DARNED (Kiran et al. 2013) databases as well as editing sites identified in this study were used. From this repertoire of editing sites, 68 sites (33 novel sites, this study) were found in the 9-base-long 5′ ss, and 66 sites (25 novel sites, this study) were found in the 23-base-long 3′ ss sequence. MaxEnt scores were obtained for both the edited and unedited versions of the sequence, and then the difference in MaxEnt scores was calculated (Supplemental Table S5). The majority of editing sites in the 5′ splice site reduces the MaxEnt scores, indicating that A-to-I editing reduces the strength of the 5′ splice sites. In contrast, in eight cases (four novel sites identified here), editing leads to 5′ splice site creation along with a strong improvement of the MaxEnt score (Supplemental Table S5). On the contrary, editing in the 3′ splice site does not follow any particular trend except in 11 cases (four novel sites from this study) where editing leads to 3′ splice site disruption (AG → GG) accompanied by a consistent decrease in MaxEnt score (Supplemental Fig. S11). We validated the effect of editing on splicing for five different editing sites using a mini-gene approach. The difference in the MaxEnt scores correctly predicted the impact on splicing in all five cases (Supplemental Fig. S12). Overall, this led us to conclude that A-to-I editing events in the bases surrounding splice sites impacts the strength of splice sites, frequently leading to a reduction in splicing.

### ADARB1-mediated filamin, alpha editing causes intron retention

The differential editing analysis performed in this study confirmed that filamin, alpha (*Flna*) is an ADARB1 target (Stulić and Jantsch 2013). *Flna* gets edited at exon 42 where CAG → CGG conversion leads to recoding of glutamine (Q) to arginine (R).

**Figure 4.**
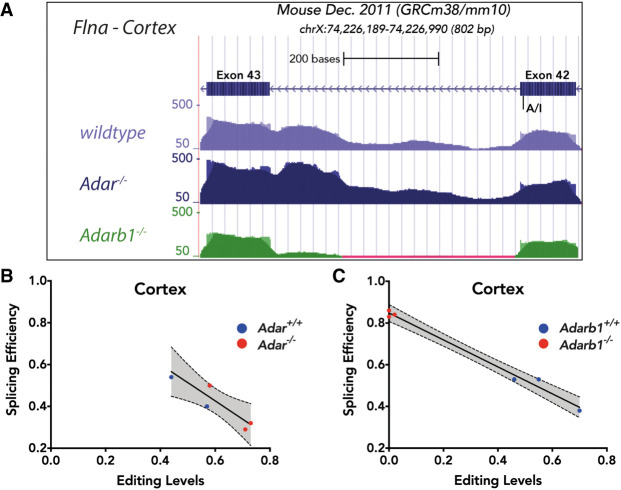
ADARB1-mediated filamin, alpha (*Flna*) pre-mRNA editing causes intron retention. (*A*) RNA-seq coverage profile of *Flna* locus at exon 42-exon 43 from WT, *Adar* KO, and *Adarb1* KO cortices. The editing site is shown at exon 42. (*B*,*C*) Linear regression of co-occurrence analysis comparing editing levels and splicing levels of *Flna* in *Adar* KO (*B*) and *Adarb1* KO (*C*) cortex. WT samples are shown in blue and KO samples are shown in red.

In the cortex of *Adar* KO mice, *Flna* is still edited by ADARB1. Indeed, a similar number of reads spanning the editing site and adjacent intron 42 can be found in RNA-seq data of *Adar* KO and WT samples. However, in the *Adarb1* KO cortex, *Flna* is not edited. Consistently, intron 42 is alternatively spliced in the DEXSeq data (Supplemental Dataset 3), resulting in a reduced coverage of intron 42 in the knockout (Fig. 4A). This indicates that editing of *Flna* at exon 42 leads to increased retention of intron 42. Since *Flna* is primarily targeted by ADARB1, the co-occurrence analysis of *Flna* in the cortex shows highest splicing efficiency in *Adarb1* KO but not in *Adar* KO (Fig. 4B,C).

### ADAR can impact splicing in an editing-independent manner

The analysis of *Adar*^*−/−*^ (*Adar*^Δ7-9^) tissues demonstrated that ADAR can influence splicing. However, in the DEXSeq-generated catalog of differential exon/intron usage events, only 129 out of 4113 in cortex, seven out of 764 in bone marrow, and 0 out of 222 in liver events were found to harbor differential editing sites in a genomic region of 5000 nt surrounding the event at the pre-mRNA-level. This suggests that the majority of differential exon/intron usage events were devoid of differential editing sites in all tested *Adar*-KO tissues. Thus, ADAR may impact their splicing in an editing-independent manner. Given that the majority (∼80%) of editing sites in the mouse can be found in intronic regions (Licht et al. 2019b), the lower coverage of intronic regions in the poly(A) RNA-seq performed in this study may partially explain this observation.

Still, to further explore this observation and to test for an editing-independent impact of ADAR on splicing, a previously published RNA-seq data set generated from 12-wk-old mouse brain isolated from mice expressing a catalytic dead (*Adar*^E861A/E861A^) version was analyzed (Heraud-Farlow et al. 2017). Embryonic lethality of these mice was rescued by concomitant deletion of *Ifih1* (Liddicoat et al. 2015). Conceptually, ADAR^E861A^ fails to edit but should still be able to bind RNA. Of note, the RNA-seq libraries of *Adar*^E861A/E861A^ mice were prepared from ribo-minus RNA and sequenced in 75-bp paired-end mode using the Illumina NextSeq 500 (Supplemental Fig. S1A,B).

**Figure 5.**
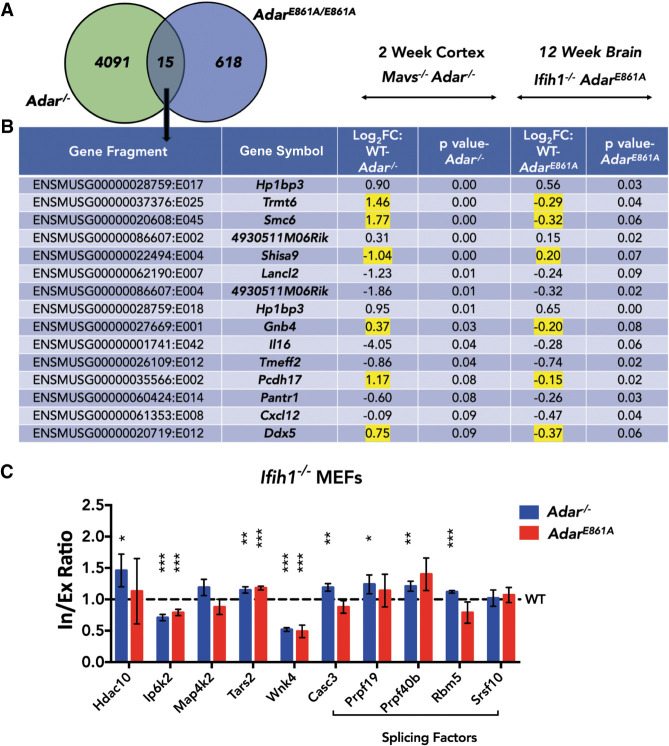
ADAR can impact splicing in an editing-independent manner. (*A*) Venn diagram comparing DEXSeq output of *Adar*^*−/−*^ cortex and editing-deficient *Adar*^E861/E861A^ brain. (*B*) Table of DEXSeq events common between *Adar*^*−/−*^ cortex and *Adar*^E861/E861A^ brain. Genes with opposite trends in the direction of change are highlighted in yellow. Log_2_FC > 0 indicates higher quantity in WT and Log_2_FC < 0 indicates higher quantity in knockout. (*C*) Histogram showing qPCR validation of splicing events in IFIH1-rescued MEFs. Data shown are mean inclusion to exclusion ratio in *Adar*^*−/−*^ and *Adar*^E861A/E861A^ (±SD) relative to *Adar*^*+/+*^. Statistical test performed was Student's *t*-test; (*) *P* < 0.05, (**) *P* < 0.01, (***) *P* < 0.001.

Analysis of the *Ifih1*^−/−^ ; *Adar*^+/+^ and *Ifih1*^−/−^ ; *Adar*^E861A/E861A^ data sets using DEXSeq identified 633 significant (*P* ≤ 0.1) differential exon or intron usage events in 448 genes in the editing-deficient *Ifih1*^−/−^; *Adar*^E861A/E861A^ brain (Supplemental Dataset 3). This suggests that the editing activity of ADAR affects some splicing events. However, the internal truncation allele used here, *Adar*^Δ7-9^, that affects RNA binding and most likely also protein stability, impacts many more splicing events. It thus appears that RNA-binding of ADAR has a strong impact on RNA-splicing. Comparison of the DEXSeq output from *Adar*^*−/−*^ cortex and *Adar*^E861A/E861A^ brain found only 15 events (*P* ≤ 0.1) in 13 genes that were common in both outputs (Fig. 5A,B). Genes in this list encode for tRNA modifying enzyme (*Trmt6*), RNA helicase (*Ddx5*), component of AMPA receptor complex (*Shisa9*), guanine nucleotide binding protein (*Gnb4*), interleukin 16 (*Il16*), or heterochromatin binding protein (*Hp1bp3*). In this list of genes, six DEXSeq events in *Trmt6, Smc6, Shisa9, Gnb4, Pcdh17*, and *Ddx5* showed opposite directions of change in *Adar*^*−/−*^ and *Adar*^E861A/E861A^. Of these, *Shisa9* and *Gnb4* have an editing site overlapping or next to the differentially spliced site. Thus, an impact of RNA-editing and RNA-binding on RNA-splicing seems possible. The small overlap between *Adar*^*−/−*^ cortex and *Adar*^E861A/E861A^ brain samples may also result from different sublocalized gene expression patterns (in cortex vs. whole brain), age (2 wk vs. 12 wk), different RNA-seq read depth (7 × 10^7^ 125-bp PE vs. 7 × 10^7^ 75-bp PE), and library preparations (ribo-minus vs. poly[A]) (Supplemental Fig. S1A,B).

In any case, to test for an editing-independent impact of ADAR on splicing, we used mouse embryonic fibroblasts (MEFs) generated from IFIH1-rescued wild-type (*Ifih1^−/^ ; Adar*^*+/+*^), knockout (*Ifih1^−/−^ ; Adar*^*/−*^), and knock-in (*Ifih1^−/−^ ; Adar*^E861A/E861A^) mice. Candidates that were already validated in the MAJIQ/DEXSeq data sets in the *Adar* KO cortex and that were expressed in the MEFs were evaluated by qPCR (Fig. 5C). Eight out of 10 targets were differentially spliced to significant extents in either *Adar*^*−/−*^ or in both *Adar*^*−/−*^ as well as *Adar*^E861A/E861A^ MEFs. Five substrates, *Hdac10*, *Casc3*, *Prpf19*, *Prpf40b*, and *Rbm5*, showed impaired splicing only in *Adar*^*−/−*^ and not in *Adar*^E861A/E861A^, suggesting that splicing in these targets is mediated by ADAR in an editing-independent manner. On the contrary, *Ip6k2*, *Tars2*, and *Wnk4* showed impaired splicing in both *Adar*^*−/−*^ as well as in *Adar*^E861A/E861A^, suggesting that the editing activity of ADAR is relevant. Overall, this led us to conclude that ADAR can impact splicing via editing-dependent and editing-independent pathways.

### ADAR-mediated impact on splicing affects circular RNA biogenesis

Circular RNAs (circRNAs) are formed by back-splicing of exons or introns, and their biogenesis is also mediated by the spliceosome (Memczak et al. 2013). ADAR has been implicated in circRNA biogenesis, and *ADAR* knockdown leads to enhanced circRNA expression in human cells (Memczak et al. 2013; Ivanov et al. 2015; Rybak-Wolf et al. 2015). However, a tissue-specific analysis in mice lacking *Adar* was not performed to this point. As circular RNAs lack poly(A) tails, the ribosomal-RNA-depleted RNA-seq performed on bone marrow and liver data sets seemed suitable for this analysis (Bajad et al. 2020). The data of WT and *Adar* KO tissues were compared for expression profiles of circRNAs using the DCC workflow (Cheng et al. 2016). The program systematically detects back-spliced junctions from RNA-seq data. We found 1825 and 1768 circRNAs in the bone marrow and liver data sets, respectively (Supplemental Dataset 6). Subsequently, read counts from both linear reads as well as circular (back-spliced) reads obtained from DCC were used to perform differential expression analysis using edgeR (Supplemental Fig. S13A,B). Out of 1825 detected, 242 circRNAs and 318 (out of 1768 detected) circRNAs were differentially expressed (*P* ≤ 0.05) in bone marrow and liver, respectively. Next, we searched for circRNAs that were differentially expressed irrespective of their host gene expression. In bone marrow, 197 (out of 242) circRNAs differed significantly only in their circular counts, whereas 45 circRNAs were significant in both circular counts as well as linear counts. In liver, 298 (out of 318) circRNAs differed significantly only in their circular counts, whereas only 20 circRNAs differed in both circular as well as linear counts. Among all the circRNAs detected, 1498 circRNAs were commonly expressed in both bone marrow and liver data sets. However, none of the significantly changed circRNAs in bone marrow showed significant changes in liver and vice versa.

Since the majority of the significantly differentially expressed circRNAs showed no altered expression of their linear counterparts, ADAR seems likely involved in their biogenesis. To obtain mechanistic insights for ADAR-mediated circRNA biogenesis, the regions flanking the circRNA coordinates were scanned for an enrichment of editing sites. However, given the currently known editing sites and sites detected in this study, no such enrichment was found in either bone marrow or liver circRNAs. Also, the average gene expression of host genes from which significant circRNAs emerged was not different from the expression of host gene from which average circRNAs were derived (Supplemental Fig. S13C).

As ADAR has an impact on alternative splicing and since back-splicing is a type of alternative splicing, we next asked if the ADAR-mediated impact on alternative splicing can in turn perturb circRNA biogenesis. To this end, we intersected circRNA coordinates obtained from DCC analysis with differential exon or intron usage events obtained from DEXSeq analysis. Here, of 1825 circRNAs detected in the bone marrow data set, 139 overlapped with DEXSeq genes. Of these 139 circRNAs, nine overlapped exactly with the coordinates of differential exon/intron usage coordinates (Supplemental Table S6). Similarly, out of 1768 detected circRNAs in the liver data set, 47 overlapped with DEXSeq genes in liver. Of these 47 circRNAs, three overlapped exactly with the coordinates of differential exon/intron usage coordinates (Supplemental Fig. S13D; Supplemental Table S6). Although a mere overlap of coordinates may not necessarily suggest an impact on circRNA biogenesis, it implies that these loci are hotspots of circRNA biogenesis. CircRNA biogenesis may be influenced by closely spaced paired repeat elements like inverted SINEs. Indeed, the set of circRNAs differentially expressed in bone marrow but not in liver was closer to downstream paired SINE elements (Supplemental Fig. S14A–C). Similarly, the distance to downstream flanking exons was closer for circRNA differentially expressed in the bone marrow but not in the liver (Supplemental Fig. S14D–F). Overall, this analysis detected differentially expressed circRNAs in *Adar* KO tissues, suggesting that ADAR may affect a few selected circRNAs in their biogenesis.

## Discussion

The cotranscriptional nature of mRNA processing has permitted evolution of various coupling mechanisms such as RNA editing and pre-mRNA splicing.

In this study, using genetic mouse models in which either one of the two catalytically active editing enzymes ADAR or ADARB1 are deleted, we determined their impact on pre-mRNA splicing. So far, the study of splicing in *Adar*-ablated mouse postpartum was precluded due to embryonic lethality. Our study fills this gap by using *Adar*-deficient mice that are rescued by a deletion of *Mavs*. We compared these data with *Adarb1*-deficient mice rescued by a pre-edited *Gria2* allele (Higuchi et al. 2000). We compared transcriptome-wide editing patterns and splicing changes in *Adar*- and *Adarb1*-ablated mouse tissues using the bioinformatics tools MAJIQ and DEXseq. MAJIQ only picked up a small number of alternative splicing events, leaving out events that were already known to be regulated by RNA editing (Higuchi et al. 2000; Flomen et al. 2004; Schoft et al. 2007; Licht et al. 2016). To overcome this shortcoming, we combined MAJIQ with an altered version of DEXSeq (Anders et al. 2012). Doing so allowed us to identify a total of 3573 genes with altered exon/intron usage patterns.

Previously, we had identified around 90,000 editing sites in the mouse transcriptome, the majority of which are located in intronic regions (Licht et al. 2019b). Consistently, many of the alternatively spliced genes identified here are predicted to harbor one or more editing sites. Nevertheless, the majority of these events were not differentially edited upon loss of ADAR or ADARB1, as can be seen here: https://genome.ucsc.edu/ cgi-bin/hgTracks?hgS_doOtherUser=submit&hgS_otherUserName=utkarshkapoor87&hgS_otherUserSessionName=Editing%2FSplicing%20Interplay.

A concern is the ∼25% false discovery rate of alternative splicing events. This reflects the shortcoming of available tools to identify alternative splicing patterns. These tools are unable to detect the entire complexity of mammalian transcriptomes, as pointed out before (Liu et al. 2014). Accordingly, the overlap between MAJIQ and DEXseq events is relatively small. Also, the false discovery rate is comparable for both ADAR and ADARB1 target sites.

In fact, we found only 23 genes that harbored 37 differential editing sites overlapping the coordinates of the DEXSeq event (i.e., the alternatively spliced region) in *Adar* KO cortex. However, the detection of differentially edited sites requires high coverage. Thus, we may have missed several differentially edited sites in particular in intronic regions of transcripts. Still, in a window of 5000 nt up- or downstream from the spliced region, a total of 207 differentially edited sites were found; harbored in 129 splicing events predicted by DEXSeq in *Adar*-deficient cortex. The relatively small number of differentially edited sites in the vicinity of differentially spliced regions suggests that the majority of the adenosine deaminase-mediated impact on splicing may be indirect and act in *trans*. In fact, genes alternatively spliced in the *Adar* KO cortex were frequently associated with the GO terms “mRNA-splicing via spliceosome,” “mRNA-processing,” and “gene expression.” This supports the idea that most altered splicing events are mediated by altered expression patterns of genes affecting RNA metabolism. Consistent with a previous study (Solomon et al. 2013), this suggests that ADAR majorly impacts the global splicing landscape by altering the splicing pattern of *trans*-acting splicing factors.

Our analysis revealed differences in the splicing landscape in the *Adar* KO tissues cortex, bone marrow, and liver. We found that the absence of *Adar* can impact mRNA-splicing in a tissue-specific manner, both in terms of trend and magnitude. This may be regulated by affecting different, tissue-specific splicing factors, by tissue-specific ADAR interactomes, inosine reader proteins, or even changes in transcriptional kinetics. In three different tissues, no single common editing target was edited and/or spliced to explain the global changes in splice patterns observed. The tissue-specific impact of ADARB1-mediated RNA editing on pre-mRNA splicing remains to be tested. Given that editing sites are edited in a tissue-specific manner, it would be interesting to test tissue-specific splicing outcomes of nonsynonymous editing targets that are recoded as a result of ADARB1 editing.

In order to enrich for genes that are directly impacted by editing, we developed an orthogonal approach where we analyzed co-occurrence of editing and splicing events in individual reads. We observed a reduction in splicing efficiency of recoding editing targets like *Flna, Flnb, Gria2, Mfn1*, and *Tmem63b*. Since this approach was limited to a ±50-nt window, an even higher number of editing sites that impact splicing events might be detected by increasing the scanning window. Furthermore, it remains to be tested if the editing-splicing interplay impacts specific RNA-isoforms where third generation single molecule sequencing technologies and long reads may come in handy (McCarthy 2010; Jain et al. 2016).

A particularly interesting editing target is *Flna* which is edited throughout multiple tissues to different extents (Stulić and Jantsch 2013). Our data provide evidence that RNA editing of *Flna* can reduce its splicing efficiency, supporting the prediction from co-occurrence analysis. It would be interesting to test if the *Flna* editing-splicing link has any physiologically relevant effect in different tissues given that *Flna* is edited to different levels in different tissues (Stulić and Jantsch 2013).

We found that the majority of genes with altered splice patterns identified in this study did not harbor any editing sites that were differentially edited upon either *Adar* or *Adarb1* deletion. We therefore tested for the impact of ADAR on splicing in an editing-independent manner. By comparing the splicing landscape between IFIH1-rescued *Adar* WT and *Adar*^E861A/E861A^ mouse brain (Heraud-Farlow et al. 2017), we were able to uncover 448 genes that showed altered splicing patterns in *Adar*^E861A/E861A^. However, an IFIH1-rescued *Adar* KO data set, an important control supporting our analysis, was missing in Heraud-Farlow et al. (2017). Therefore, a three-way comparison of global splicing landscapes between *Adar* WT, *Adar* KO, and *Adar*^E861A/E861A^ in the same tissue remains to be tested. Nevertheless, our data suggest that ADAR also influences alternative splicing in an editing-independent manner, likely via binding to pre-mRNAs and competing with splicing factors as we had previously observed for ADARB1 (Licht et al. 2016). Splicing can also influence editing levels via affecting the kinetics of forming and destroying editing-competent structures on the pre-mRNA level (Licht et al. 2016, 2019b). These dynamics may be modulated by alternative splicing factors (Licht et al. 2019b; Quinones-Valdez et al. 2019). Overall, our study using connected, yet independent orthogonal approaches demonstrates a widespread but predominantly indirect effect of ADAR on splicing. In contrast, ADARB1 affects splicing to a much lower extent but acts seemingly more directly by altering regulatory sequences in the immediate vicinity of 5′ and 3′ splice-sites.

Lastly, we analyzed changes in circRNA expression upon *Adar* knockout in bone marrow and liver. The impact of ADAR on circRNA expression was previously established using siRNA-mediated knockdown in HEK293, SH-SY5Y, and mouse P19 cells (Ivanov et al. 2015; Rybak-Wolf et al. 2015). However, although the previous data suggest a general up-regulation of circRNA expression upon loss of ADAR, we observed distinct differences for individual circRNAs including both up- and down-regulation. Moreover, in our analysis, changes in circRNA expression upon *Adar* loss correlate with the expression changes seen for their linear counterparts.

Together with a comprehensive analysis of circRNA genesis in the presence or absence of ADARs, our study provides a global and comprehensive view on the interplay of editing, RNA splicing, and circRNA biogenesis. Our integrated and comprehensive data set is available as a resource in the form of a UCSC Genome Browser session titled “Editing-Splicing Interplay.”

## Methods

### RNA-seq

*Mavs*^*+/−*^ mice were acquired from Jackson Laboratory (stock #008634, Allele: *Mavs*^tm1Zjc^). *Adar*^Δ7-9^ and *Adarb1^+/−^; Gria2*^R/R^ were kindly provided by Dr. Peter Seeburg (Higuchi et al. 2000; Hartner et al. 2004). Mice were bred using standard in-house mouse facility/FELASA guidelines. For RNA-seq, age-/sex-matched littermate mice of desired genotypes were sacrificed at 2 wk of age, the cortex was isolated, and RNA was extracted using TriFast (VWR Peqlab) and DNase I treated (Thermo Fisher Scientific) following the manufacturer's instructions. Three biological replicates were used for each genotype. To prepare RNA-seq libraries, we started with 1 µg total RNA from each sample and performed poly(A) RNA selection using the NEBNext Poly(A) mRNA Magnetic Isolation Module (New England Biolabs). cDNA libraries were subsequently generated from isolated poly(A) RNA using the NEBNext Ultra Directional RNA Library Prep kit for Illumina (New England Biolabs), barcoded using NEBNext Multiplex Oligos for Illumina Index Primers Set 1 (New England Biolabs), and sequenced in a paired-end mode with 125-bp read length using the HiSeq 2500 (Illumina) platform.

### Differential editing analysis

Differential editing analysis was performed by comparing editing levels in WT and KO samples. Only those editing sites were considered for analysis that were covered by minimum five reads in at least two out of three WT and in at least two out of three KO samples. We then compared the mean of editing levels of sites in WT and KO samples and performed statistical analysis using Welch's *t*-test, and all editing sites that had a *P*-value ≤ 0.1 were considered significantly differentially edited. For comparison with previously known sites, mouse editing sites were downloaded from RADAR (Ramaswami and Li 2014) and DARNED (Kiran et al. 2013) databases.

### Global splicing analysis

For profiling global splicing changes in *Adar* and *Adarb1* knockout tissues, we first used a tool called Modeling Alternative Junction Inclusion Quantification which detects and quantifies local splicing variations from RNA-seq data (Vaquero-Garcia et al. 2016). Default parameters were used. It quantifies relative abundance (PSI) of LSVs and changes in relative LSV abundance (delta PSI) between genotypes. We also used a second tool which estimates Differential Exon Usage (DEXSeq) from RNA-seq data (Anders et al. 2012). This program evaluates if a certain exon is under-/overrepresented relative to all the other exons in that gene in different experimental conditions. By default, DEXSeq excludes any intronic events. Since we know that A-to-I RNA editing events are enriched in the introns, we tweaked DEXSeq to include intron information in the input mouse (assembly GRCm38/mm10) annotation (GTF) file. RNA-seq data from *Adar*^+/+^; *Ifih1*^−/−^ and *Adar*^E861A/E861A^; *Ifih1*^−/−^ adult whole brain was retrieved from NCBI Gene Expression Omnibus (GEO) (GSE94387).

### Validation of differential splice events from MAJIQ and DEXSeq

To validate differential splicing events called in MAJIQ and DEXSeq data sets, we used an RT-PCR approach. Total RNA was extracted using TriFast (VWR Peqlab) and DNase I treated (Thermo Fisher Scientific) following the manufacturer's instructions. One microgram of DNase-treated RNA was reverse-transcribed (Thermo Fisher Scientific) using the manufacturer's instructions. NEB OneTaq Quick-Load 2X Master Mix with Standard Buffer (New England Biolabs) was used for RT-PCR with sequence-specific primers (Supplemental Table S7). Conditions for PCR are as follows: initial denaturation at 95°C for 3 min, 35 cycles of (95°C, 30 sec; annealing at 55° or 58°C, 30 sec; extension at 68°C, 30 sec), followed by final extension at 68°C for 5 min. RT-PCR products were subjected to agarose gel electrophoresis and imaged using Image Lab Software (Bio-Rad Laboratories Inc.). We also validated targets in a more quantitative manner by qPCR using NEB Luna Universal qPCR Master Mix (New England Biolabs). In this case, we used primers that specifically amplified pre-mRNA or mRNA and compared inclusion/exclusion ratio between genotypes (Supplemental Table S7). Conditions for qPCR are as follows: initial denaturation at 95°C for 1 min, 40 cycles of (95°C, 15 sec; annealing/extension at 60°C, 30 sec; extension at 68°C, 30 sec + plate read) followed by a melt curve. Splicing was quantified as inclusion to exclusion ratio and calculated as described in a previously published method (Harvey and Cheng 2016).

### Co-occurrence analysis for identification of differential splicing/editing patterns

For identification of direct targets of editing-dependent splicing variations, we used an in-house-developed method we term “co-occurrence analysis.” Here, we used the same RNA-seq data described above and looked for reads that span all known splice sites. Next, we scanned for editing sites 50 nt on either side of the exon/intron boundary. We looked for RNA-seq read combinations in a 2 × 2 matrix—unspliced-unedited, unspliced-edited, spliced-unedited, and spliced-edited—and feed these numbers in a contingency table to perform Fisher's exact test. We can also derive splice efficiency and editing levels from these reads. From this analysis, we derive the statistical probability for the co-occurrence of a splicing/editing pair.

### Circular RNA analysis

For profiling the impact of ADAR on circular RNAs, we applied the DCC pipeline (Cheng et al. 2016) on liver and bone marrow RNA-seq data from *Adar* WT and *Adar* KO (Bajad et al. 2020). These RNA-seq data sets are prepared from ribo-minus libraries making them suitable for circRNA analysis. Differential expression analysis was performed using the Degust analysis tool (https:// victorian-bioinformatics-consortium.github.io/degust/). For differential circRNA expression analysis, we used circular and linear read counts obtained from DCC as input for edgeR (Robinson et al. 2010).

## Data access

RNA-seq data generated in this study have been submitted to the European Nucleotide Archive (ENA; https://www.ebi.ac.uk/ena/browser/home) under accession numbers PRJEB31565 and PRJEB31568. Additionally, output from RNA-seq data analysis including editing sites from RDDpred, differential editing sites, LSVs from MAJIQ analysis, differential exon/intron usage events from DEXSeq, and circular RNAs from DCC have been compiled in a user-friendly UCSC Genome Browser public session named “Editing-Splicing Interplay.” The session can be accessed using the following link: https://genome.ucsc.edu/cgi-bin/hgTracks? hgS_doOtherUser=submit&hgS_otherUserName=utkarshkapoor87&hgS_otherUserSessionName=Editing%2FSplicing%20Interplay. Scripts for the co-occurrence analysis are available from GitHub (https://github.com/fabou-uobaf/ES-SS-cooccurence) and as Supplemental Code.

## Competing interest statement

The authors declare no competing interests.

## Supplementary Material

Supplemental Material
