# Supplementary material for: ADAR-deficiency perturbs the global splicing landscape in mouse tissues
Source: Genome Res. 2020 Aug;30(8):1107–18. doi: 10.1101/gr.256933.119 (PMC7462079; doi:10.1101/gr.256933.119)
Supplement: Supplemental Material [file supp_30_8_1107__index.html]

ADAR-deficiency perturbs the global splicing landscape in mouse tissues — ADAR-deficiency perturbs the global splicing landscape in mouse tissues — Supplemental Material 

# ADAR-deficiency perturbs the global splicing landscape in mouse tissues

## Supplemental Material

- Supplemental\_Material.pdf
- Supplemental\_Table\_S1.xlsx
- Supplemental\_Table\_S2.xlsx
- Supplemental\_Table\_S3.xlsx
- Supplemental\_Table\_S4.xlsx
- Supplemental\_Table\_S5.xlsx
- Supplemental\_Table\_S6.xlsx
- Supplemental\_Table\_S7.xlsx
- Supplemental\_Dataset\_S1.xlsx
- Supplemental\_Dataset\_S2.xlsx
- Supplemental\_Dataset\_S3.xlsx
- Supplemental\_Dataset\_S4.xlsx
- Supplemental\_Dataset\_S5.xlsx
- Supplemental\_Dataset\_S6.xlsx
- Supplemental\_Code.zip
