# Supplementary material for: ADAR-deficiency perturbs the global splicing landscape in mouse tissues
Source: Genome Res. 2020 Aug;30(8):1107–18. doi: 10.1101/gr.256933.119 (PMC7462079; doi:10.1101/gr.256933.119)
Supplement: Supplemental Material [file supp_gr.256933.119_Supplemental_Material.pdf]

Supplemental Material:

ADAR-deficiency perturbs the global splicing landscape in mouse tissues

Utkarsh Kapoor[1*], Konstantin Licht[1*], Fabian Amman[2], Tobias Jakobi[3], David Martin[1], Christoph Dieterich[3] and Michael F. Jantsch[1]

1. Center of Anatomy & Cell Biology,
   Department of Cell and Developmental Biology,
   Medical University of Vienna
   Schwarzspanierstrasse 17
   A-1090 Vienna, Austria

2. Institute of Theoretical Biochemistry
   University of Vienna
   Währingerstrasse 17
   A-1090 Vienna, Austria

3. Department of Internal Medicine III and Klaus Tschira Institute for Computational Cardiology,
   Section of Bioinformatics and Systems Cardiology,
   Im Neuenheimer Feld 669,
   University Hospital
   D-96120 Heidelberg, Germany

*) equal contribution

Correspondence to:
Michael F. Jantsch
Center for Anatomy and Cell Biology
Division of Cell- and Developmental Biology
Schwarzspanierstrasse 17
A-1090 Vienna, Austria
Email: Michael.Jantsch@meduniwien.ac.at
Tel: +43-1-40160 37510

Supplemental Figures 1-14

Supplemental Tables 1-7 (available as separate MS-Excel files)

Supplemental Datasets 1-6 (available as separate MS-Excel files)

Supplemental Materials and Methods

Supplemental References

A

Tissue	Sample	Genotype	No. of Uniquely Mapped Reads	No. of Multimapper Reads	Library Type	References
Cortex	WT	*Adar*$^{+/+}$; *Mavs*$^{-/-}$	59579633	4119011	poly(A); Paired-End 125 bp	This Study
	WT		70790037	3898596		
	WT		87791032	4882140		
	KO	*Adar*$^{-/-}$; *Mavs*$^{-/-}$	67590386	3728245		
	KO		107176375	4612203		
	KO		72682227	4865112		
			465609690			
Cortex	WT	*Adarb1*$^{+/+}$; *Gria2*$^{R/R}$	63624826	3812735	poly(A); Paired-End 125 bp	This Study
	WT		75771639	5276547		
	WT		47839031	3195618		
	KO	*Adarb1*$^{-/-}$; *Gria2*$^{R/R}$	63856080	3610492		
	KO		41634716	2766951		
	KO		53940497	4489321		
			346666789			
Bone Marrow	WT	*Adar*$^{+/+}$; *Mavs*$^{-/-}$	26131536	12376254	Ribo-minus; Paired-End 125 bp	Bajad, P. *et al*
	WT		32241360	3795958		
	WT		29232161	3111199		
	KO	*Adar*$^{-/-}$; *Mavs*$^{-/-}$	25493912	10715616		
	KO		25272418	2304696		
	KO		31125506	2694512		
			169496893			
Liver	WT	*Adar*$^{+/+}$; *Mavs*$^{-/-}$	18444683	7006939	Ribo-minus; Paired-End 125 bp	Bajad, P. *et al*
	WT		23071263	6242203		
	WT		26723842	5172515		
	KO	*Adar*$^{-/-}$; *Mavs*$^{-/-}$	21943050	9925991		
	KO		20697891	4942116		
	KO		20753692	3733165		
			131634421			
Brain	WT	*Adar*$^{+/+}$; *Ifih1*$^{-/-}$	78142824	5677396	Ribo-minus; Paired-End 75 bp	Heraud-Farlow, JE. *et al*
	WT		75225846	6421143		
	WT		75950504	5835784		
	E861A	*Adar*$^{E861A/E861A}$; *Ifih1*$^{-/-}$	66939167	4959013		
	E861A		74395170	5968780		
	E861A		61758273	4379633		
			432411784			

B

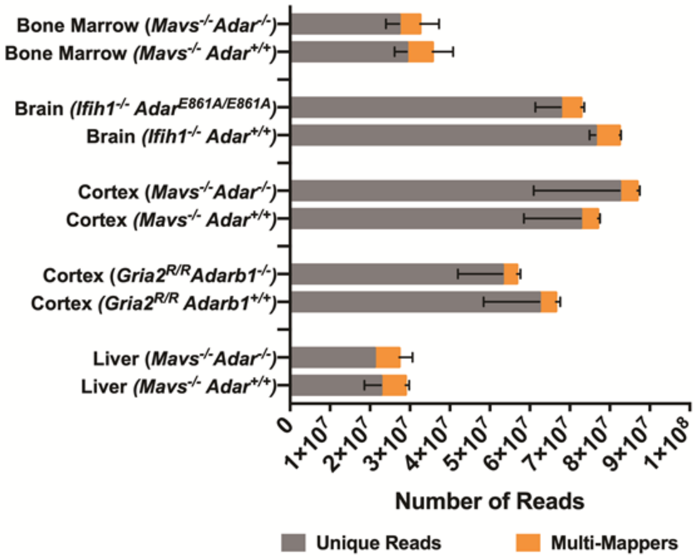

Supplemental Figure 1: <u>Overview of RNA-seq data generated and used in this study.</u> (A) Table displaying library type (poly (A) vs ribo-minus) and number of uniquely mapped reads (Heraud-Farlow et al. 2017; Bajad et al. 2020).
(B) Bar plot summarizing number of uniquely mapped reads across different ADAR and ADARB1 deficient tissues.

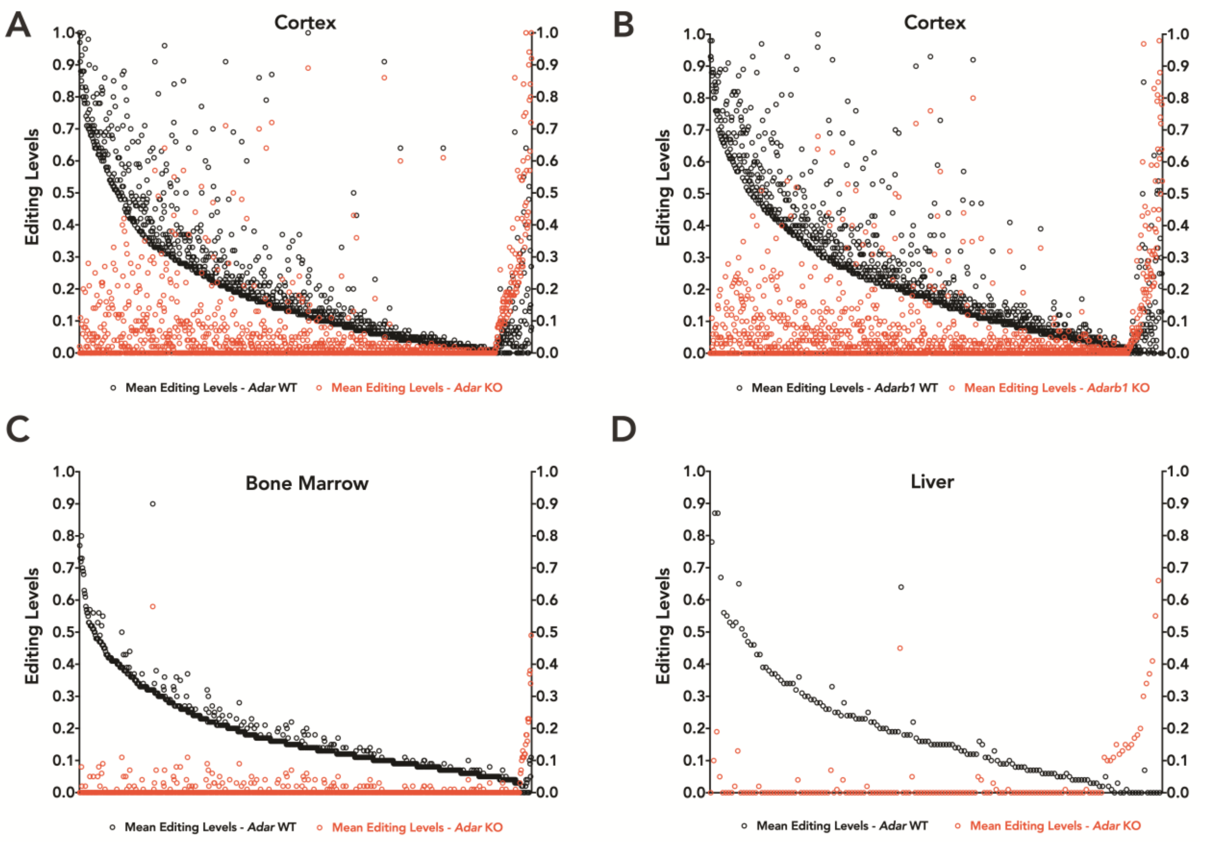

Supplemental Figure 2: <u>Mean editing levels of sites detected in different tissues of wildtype, *Adar* knockout and *Adarb1* knockout animals.</u> (A) Cortex (WT vs *Adar* KO); (B) Cortex (WT vs *Adarb1* KO); (C) Bone Marrow (WT vs *Adar* KO) and (D) Liver (WT vs *Adar* KO). Mean editing levels for every site in wildtype sample (black) and knockout sample (red) are shown. Sites have been plotted in increasing order of difference of editing levels between wildtype and mutant. Editing sites that cluster along the y-axis show an increase in mean editing levels in the mutant whereas those that cluster near the x-axis, are sites that show reduction in editing levels in the mutant.

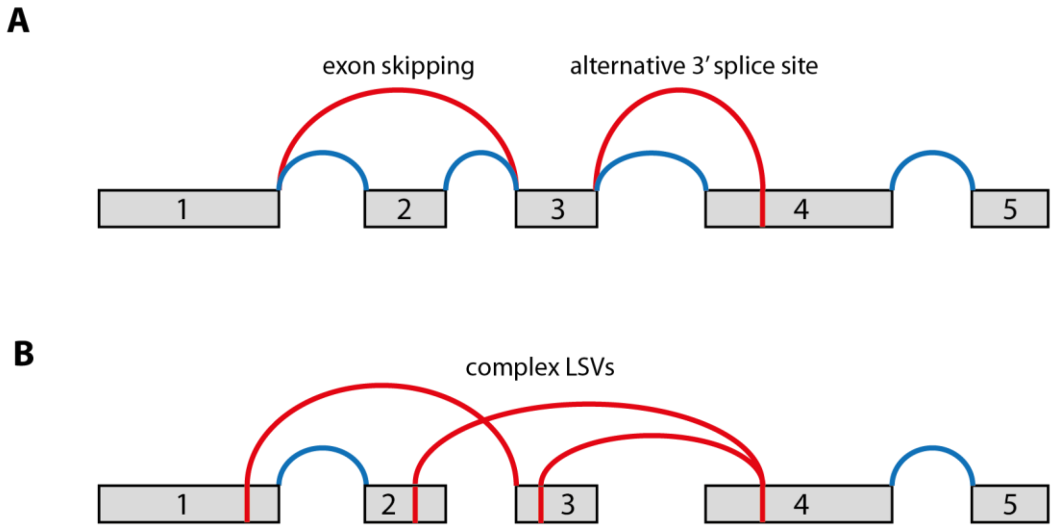

Supplemental Figure 3: Local splicing variations. (A, B) The term 'local splicing variation' (LSV) is used to describe simple and complex alternative splicing patterns. When identifying LSVs, MAJIQ mostly relies on detecting differences in 'junction reads' i.e. reads spanning two or more exons (indicated by arcs). Exons are depicted as grey bars. Red arcs indicate 'alternative splicing' whereas blue arcs indicate 'constitutive splicing'. (A) Two examples for canonical alternative splicing events are given. (B) Not all splicing events can be classified using established terminology. An example for a more complex LSV is given. The concept of LSV described in more detail in the original publication (Vaquero-Garcia et al. 2016) or online (https://majiq.biociphers.org/).

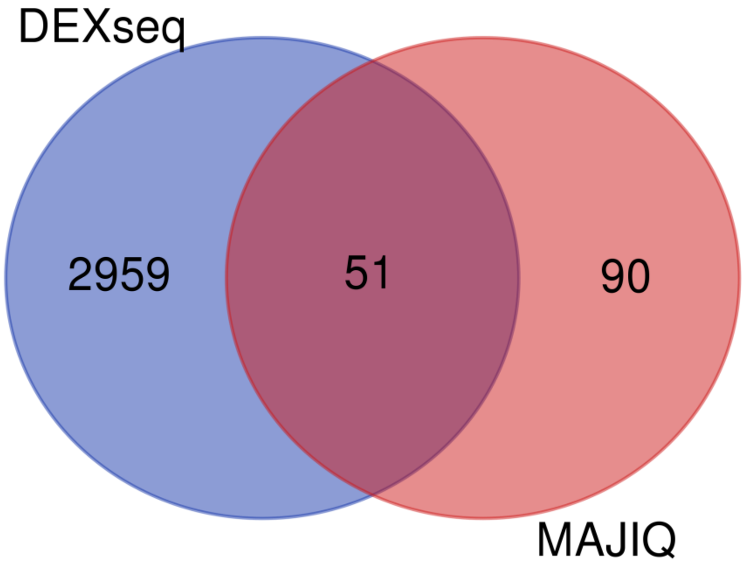

Supplemental Figure 4: Alternative splicing events detected by different methods. Venn diagram showing the overlap for the number of alternatively spliced genes in the *Adar* knockout cortex for DEXSeq and MAJIQ.

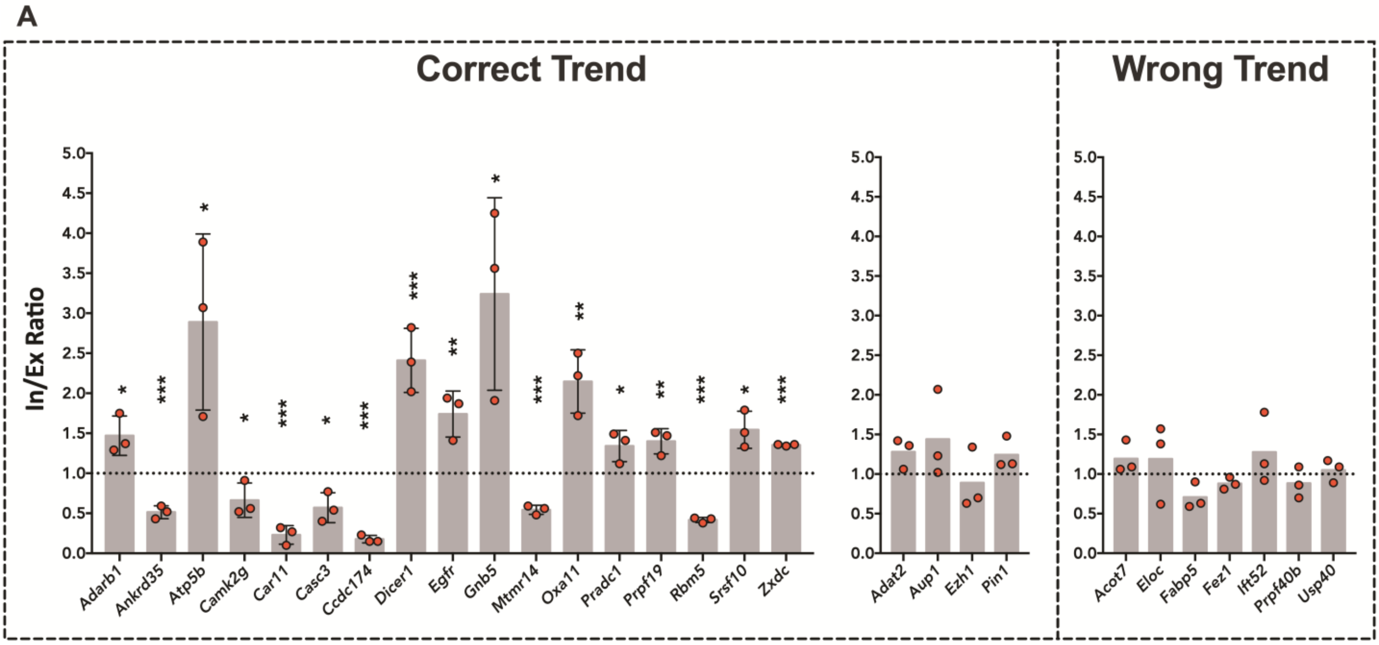

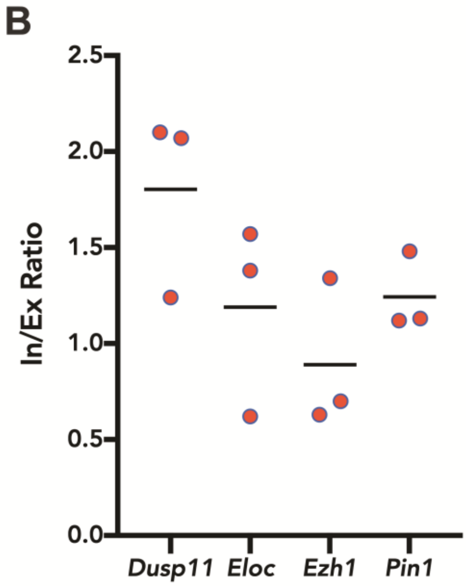

Gene	Editing Site	Editing Levels WT	Editing Levels KO	DEXSeq p-value
Dusp11	chr6:85954818	0.25	0.00	0.004
Eloc	chr1:16646834	0.14	0.00	0.012
Ezh1	chr11:101198976	0.46	0.01	0.010
	chr11:101198771	0.07	0.00	
	chr11:101198810	0.05	0.00	
Pin1	chr9:20653643	0.11	0.00	0.005

Supplemental Figure 5: qRT-PCR validations of differential exon/intron usage events predicted by DEXSeq. (A) All 29 tested targets out of which targets enclosed under “correct trend” were positively validated in all three replicates for *Adarb1*-*Zxdc* (n=3) and in at least two replicates for *Adat2*-*Pin1* (n=3). Negative validations are classified as wrong trend. (B) Validation of 4 targets which harbor differential editing sites within the coordinates of differential exon/intron usage event. *Eloc and Ezh1* are validated in at least two replicates.

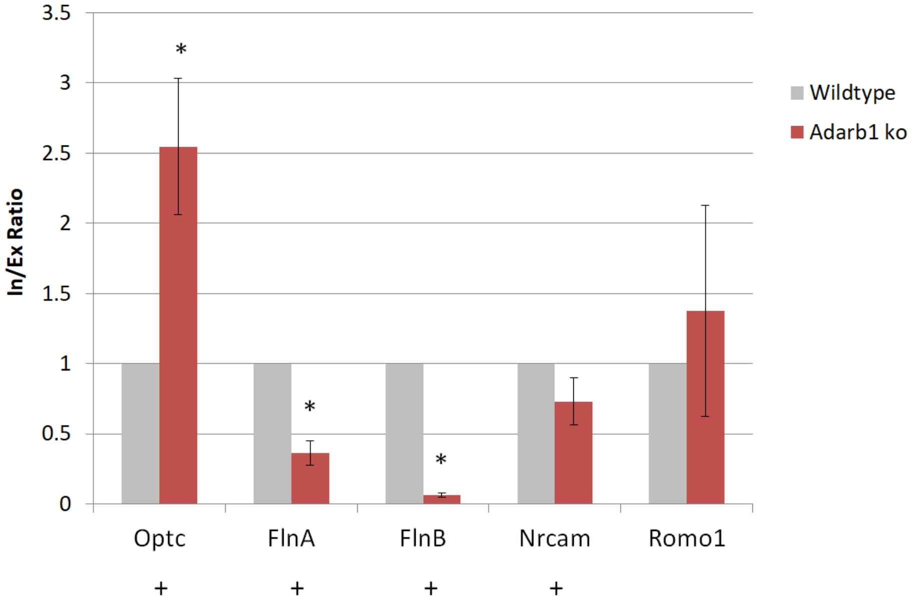

Supplemental Figure 6: qRT-PCR validations of differential exon/intron usage events in the *Adarb1* knockout cortex. Five differential splicing events predicted either by MAJIQ or DEXseq were tested using qPCR. The trend was correctly predicted for all genes except Romo1. n=3. +=correct trend. *: p-value < 0.05.

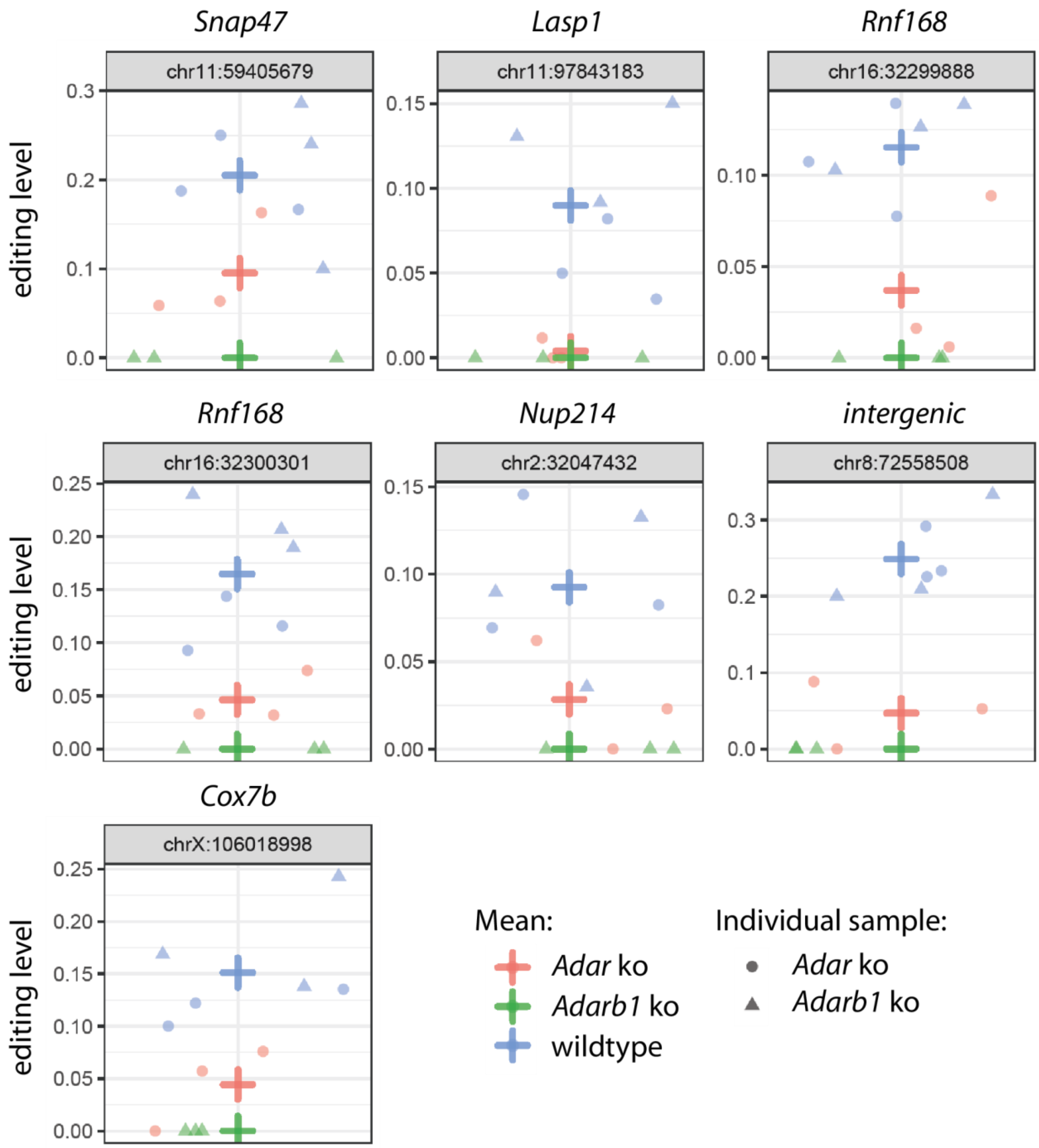

Supplemental Figure 7: <u>*Adarb1*-dependent editing sites exhibiting reduced editing upon *Adar* knockout</u>. All cortex editing sites covered by at least 10 reads in all samples were filtered according to the following criteria: editing in wildtype > 10%, no editing in the *Adarb1* ko, editing level in the *Adar* ko > than 0%, and less than 50% editing in the *Adar* ko as compared to the wildtype. The editing level for the filtered sites was plotted for all individual samples (circle: Adar, triangle: *Adarb1*), average editing levels (large crosses). The color code is depicted on the right site of the figure (blue: wildtype, green *Adarb1* ko, red: *Adar* ko). Chromosome coordinates (mm10) for all sites are given above the individual plots.

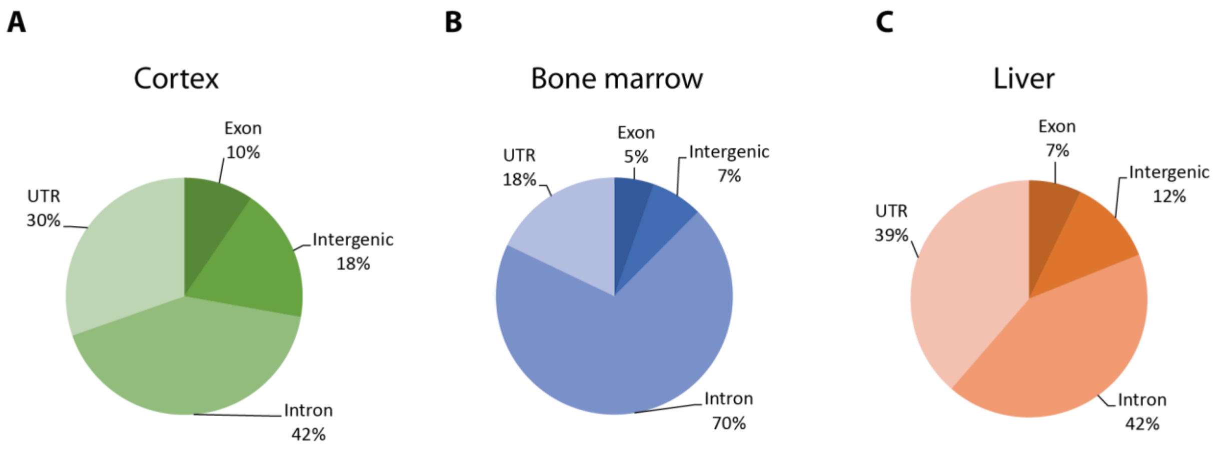

Supplemental Figure 8: Genomic annotation (Exon, Intergenic, Intron, UTR) for editing sites identified in individual tissues.

A

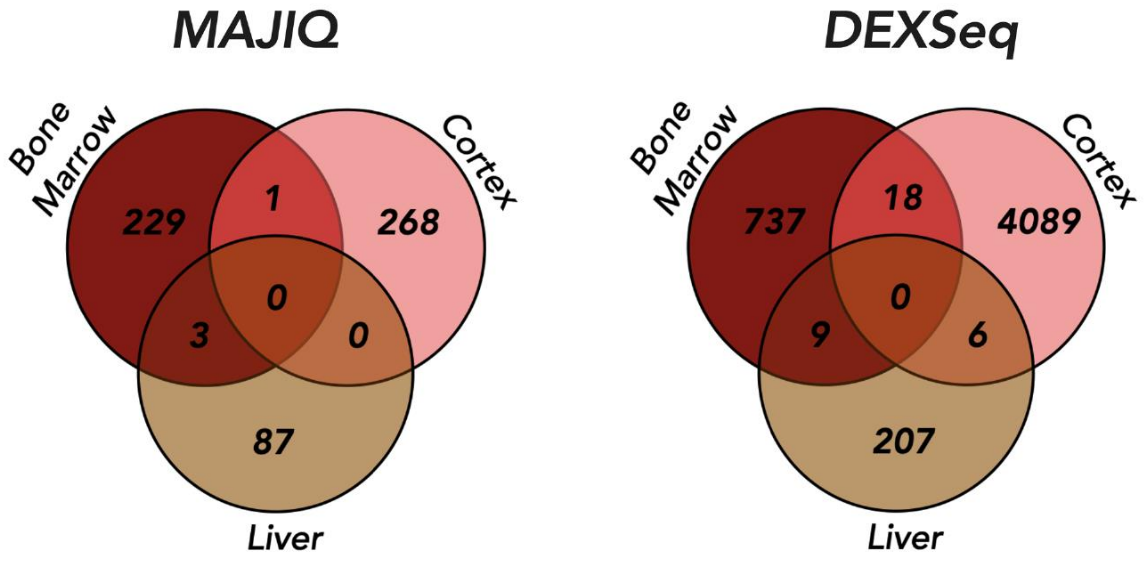

B

Gene<br>*Fragment* →	*Atp5b*<br>ENSMUSG00000025393:**E010**	*Egfr*<br>ENSMUSG00000020122:**E046**	*Fgd1*<br>ENSMUSG00000025265:**E020**	*Ttc19*<br>ENSMUSG00000042298:**E018**
Cortex	0.01	0.09	n.d.	n.d.
Bone Marrow	0.03	n.d.	0.05	~0.00
Liver	n.d.	0.01	0.08	0.04

Supplemental Figure 9: Comparison of local splicing variations across different tissues. (A) Venn diagram comparing LSV coordinates identified by MAJIQ in ADAR-deficient tissues and Venn diagram comparing differential exon/intron usage coordinates identified in ADAR-deficient tissues identified using DEXSeq. (B) Summary of adjusted p-values of targets selected for validations of differential exon/intron usage events predicted by DEXSeq in *Adar* knockout tissues.

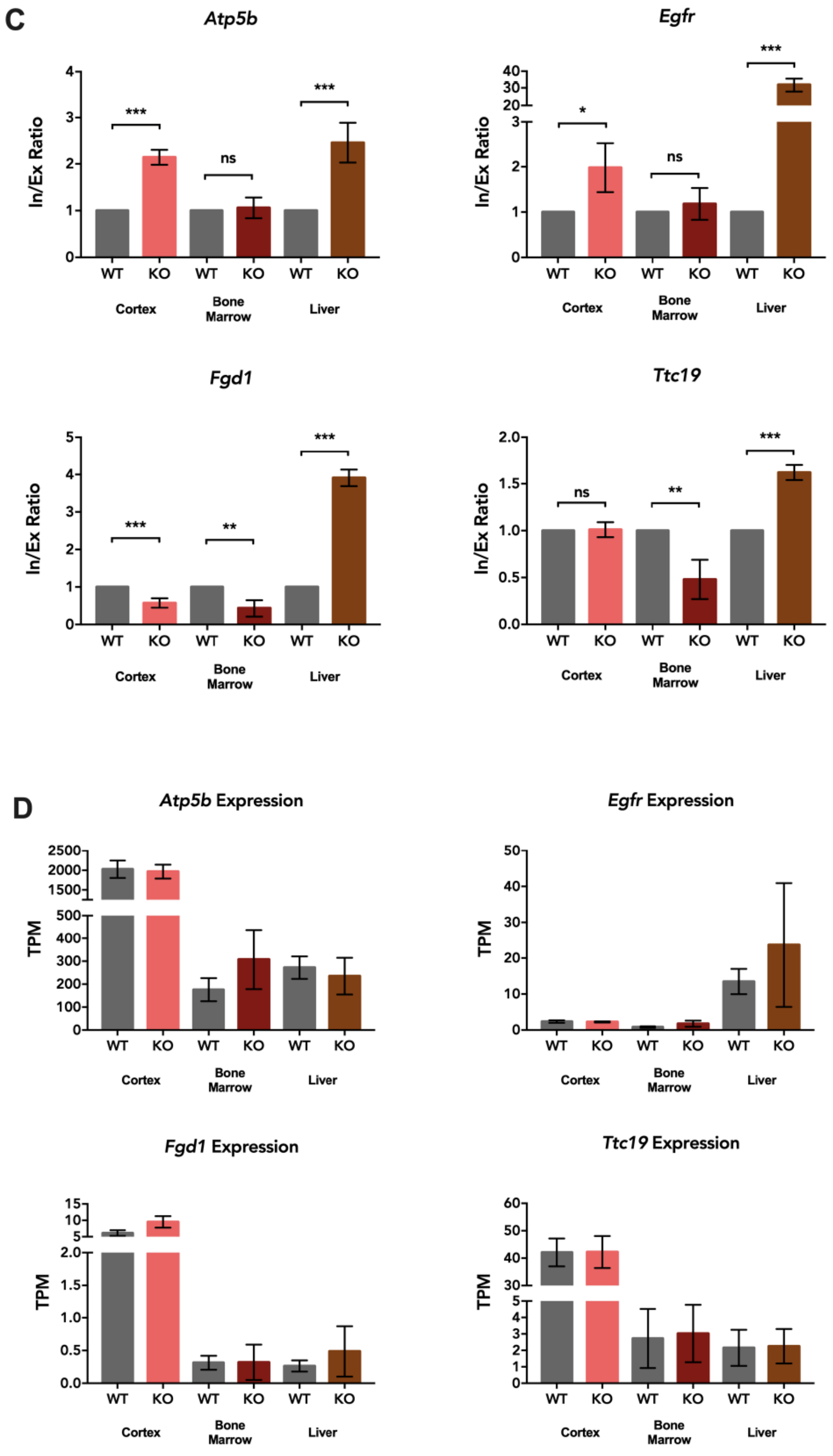

Supplemental Figure 9: <u>qRT-PCR validation of LSVs.</u> (C) Histogram showing qRT-PCR validations of LSVs in *Atp5b*, *Egfr*, *Fgd1* and *Ttc19.* Data shown is mean inclusion to exclusion ratio in *Adar* KO (+/- SD). Statistical test performed with Student's t-test (* $p < 0.05$; **: $p < 0.01$; ***: $p < 0.001$) (D) Histogram showing differential expression analysis of *Atp5b*, *Egfr*, *Fgd1* and *Ttc19* as predicted by *DESeq2*. Data shown is mean transcripts per million (TPM) values inclusion to exclusion ratio in *Adar* KO (+/- SD). None of these genes were found to be differentially expressed.

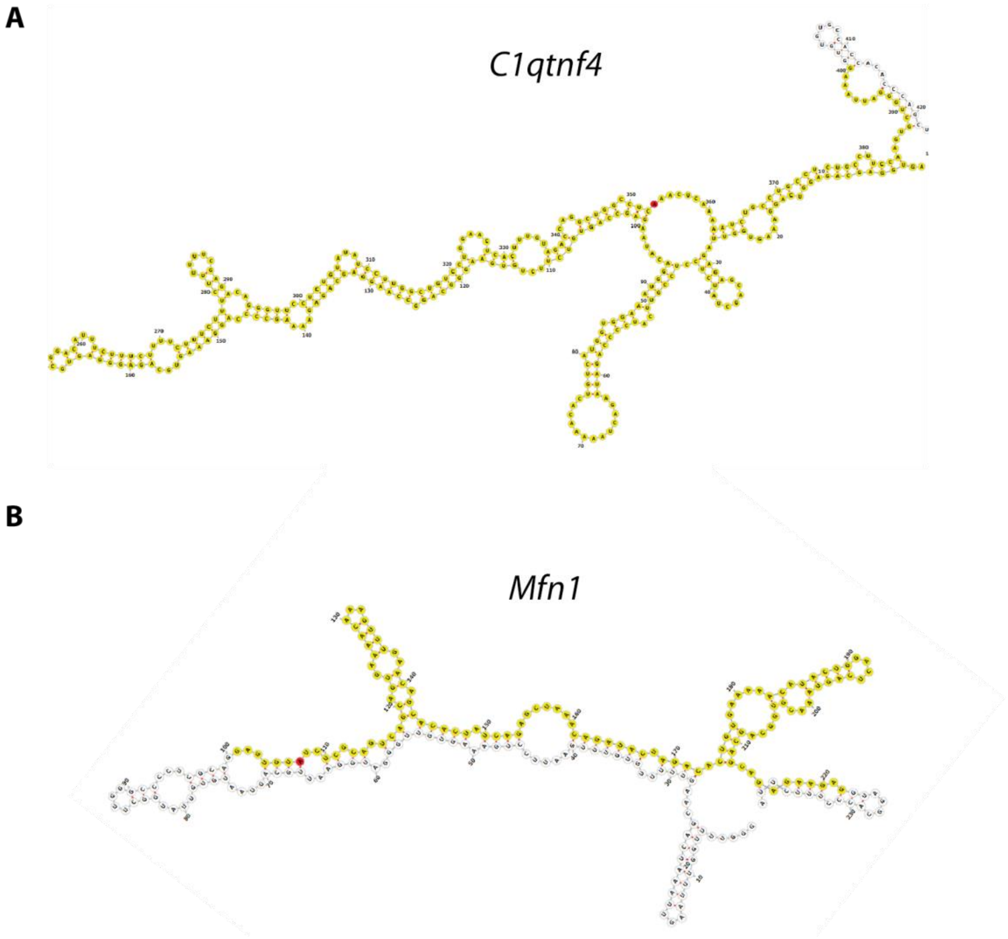

Supplemental Figure 10: Local secondary structure for two editing sites showing a strong correlation between editing and splicing. The secondary structures have been generated using the tool RNAfold in conjunction with the visualization tool forna (Lorenz et al. 2011; Kerpedjiev et al. 2015). Yellow=exon, no color=intron, red=editing site.

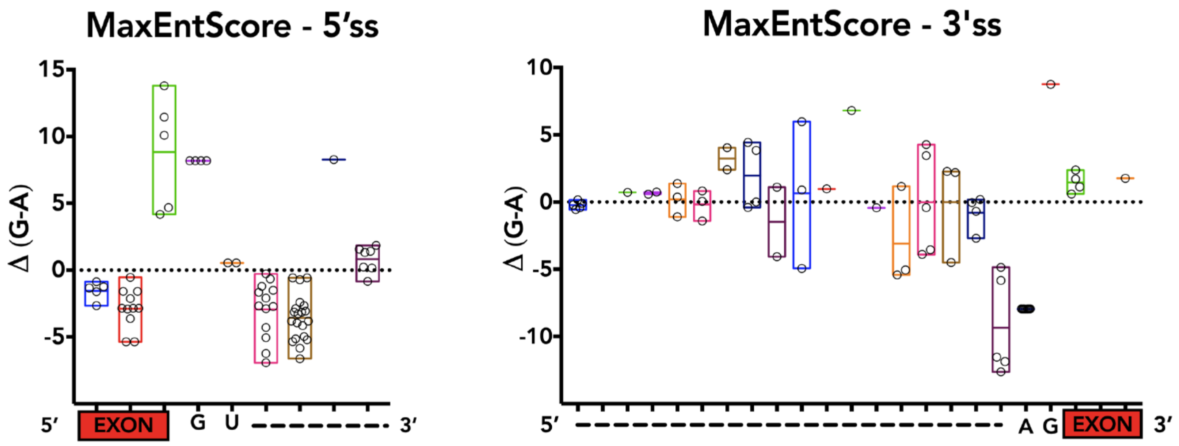

Supplemental Figure 11: Box plots depicting the change in MaxEnt scores caused by editing of sites. The Δ(G-A) score is given by position around the 5' and 3' splice sites. Each editing site is marked as a circle.

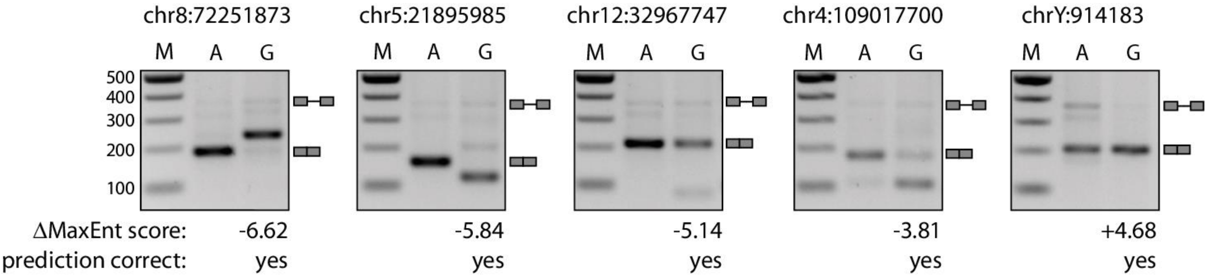

Supplemental Figure 12: Validation of splicing changes predicted by the MaxEnt score. Five editing sites (A) predicted to change pre-mRNA splicing by differences in the MaxEnt score (ΔMaxEnt) were randomly chosen and cloned into a mammalian expression vector in a heterologous context. Using site-directed mutagenesis pre-edited versions of the constructs were done (G). The constructs were transfected into Hek293T cells. Subsequently, RNA was isolated, reverse transcribed and PCR-amplified using exon-specific primers. The signals corresponding to pre-mRNA or mRNA are indicated next to each individual panel. Chromosome coordinates (mm10 are indicated on top of each panel. M=size standard.

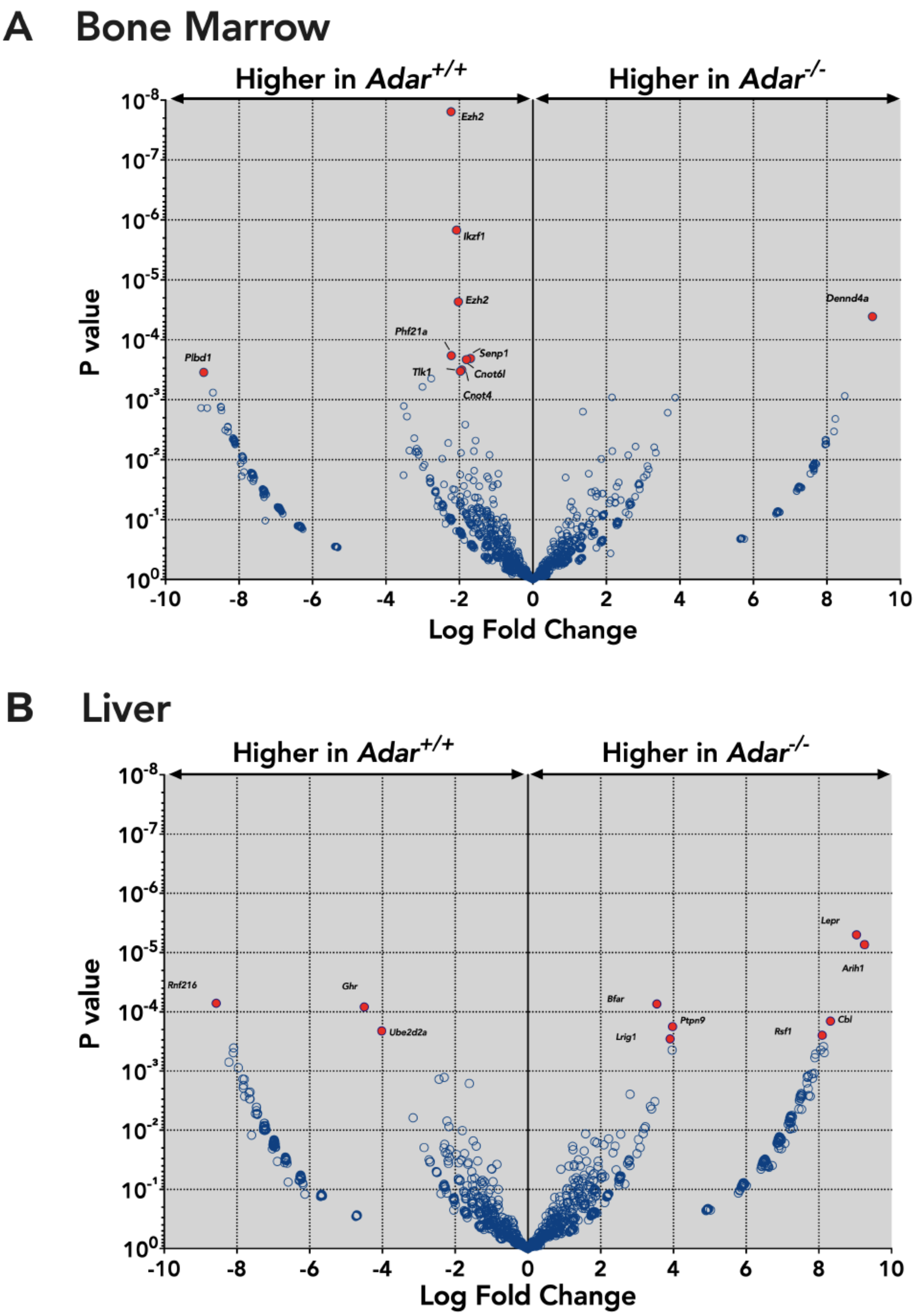
A Bone Marrow
Higher in Adar+/+
Higher in Adar-/-
P value
Log Fold Change
Ezh2
Ikzf1
Ezh2
Dennd4a
Phf21a
Senp1
Plbd1
Tlk1
Cnot6l
Cnot4
B Liver
Higher in Adar+/+
Higher in Adar-/-
P value
Log Fold Change
Lepr
Arih1
Rnf216
Ghr
Bfar
Ptpn9
Cbl
Ube2d2a
Lrig1
Rsf1

C

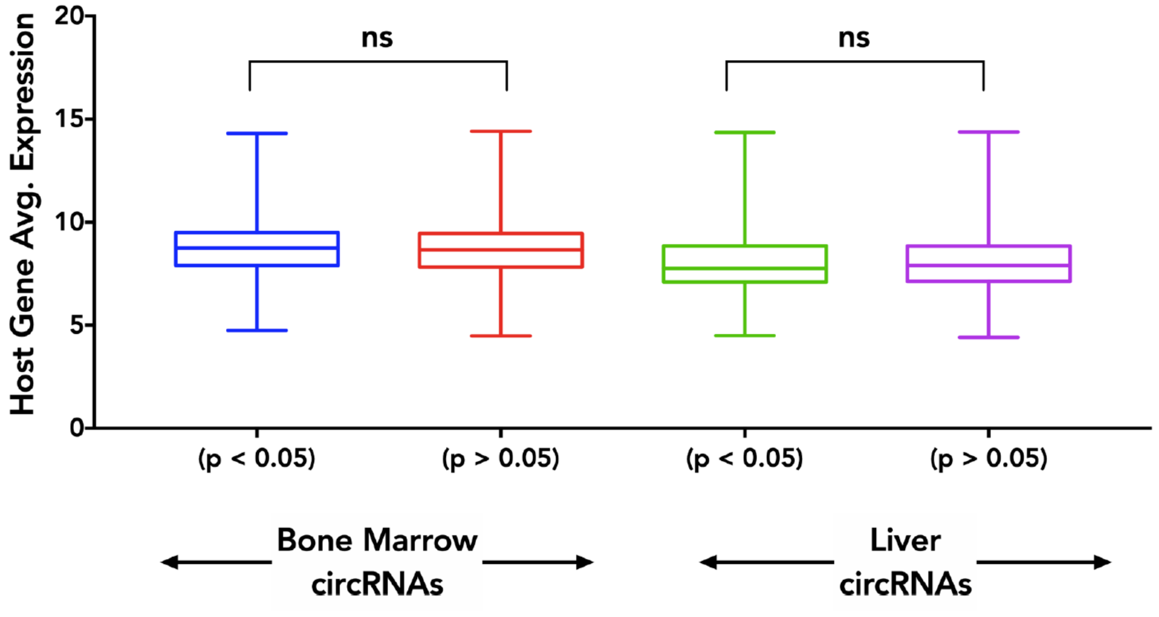

D

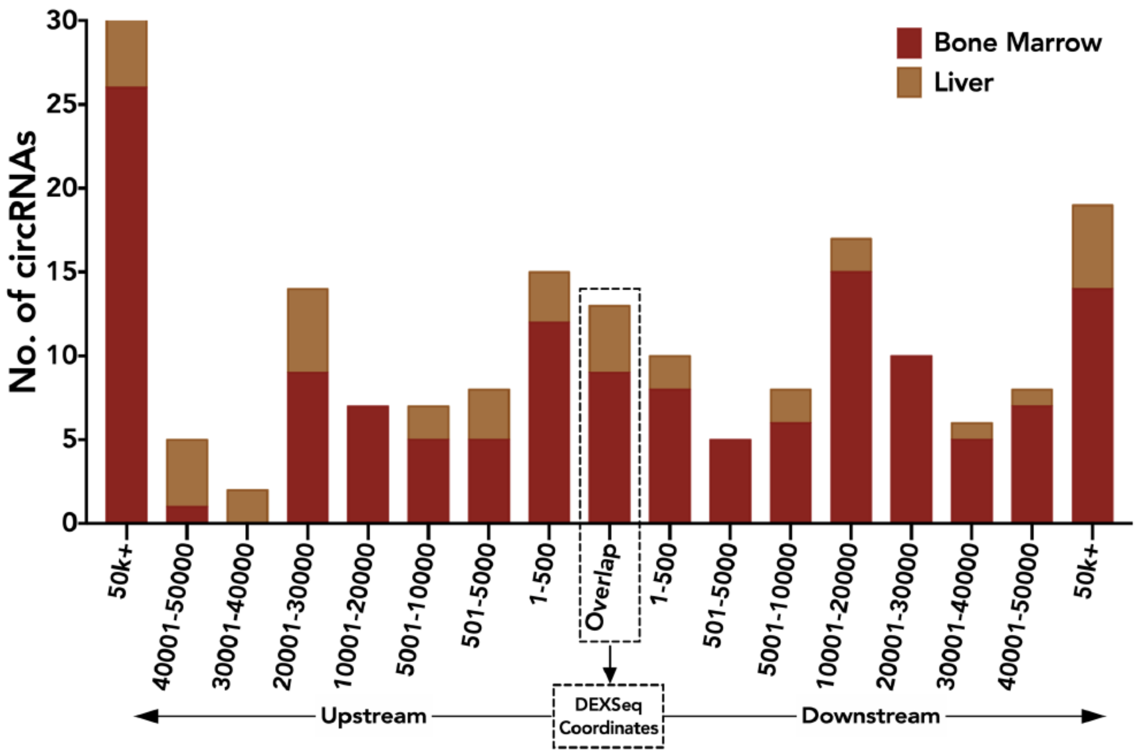

Supplemental Figure 13: Volcano plot showing differential circRNA expression in *Adar* WT and *Adar* KO bone marrow (A) and liver (B) determined using edgeR. Top 10 significant circRNAs events in both datasets are highlighted in red (C) Comparison of average host gene expression of genes that generate significantly different circRNAs ($p < 0.05$) between WT and *Adar*-KO vs. non-significant circRNAs ($p > 0.05$). (D) Histogram showing overlay of circRNAs with their host genes that were found to have a differential exon/intron usage by DEXSeq in bone marrow and liver. Circular RNAs are binned by nucleotide distance within the DEXSeq gene.

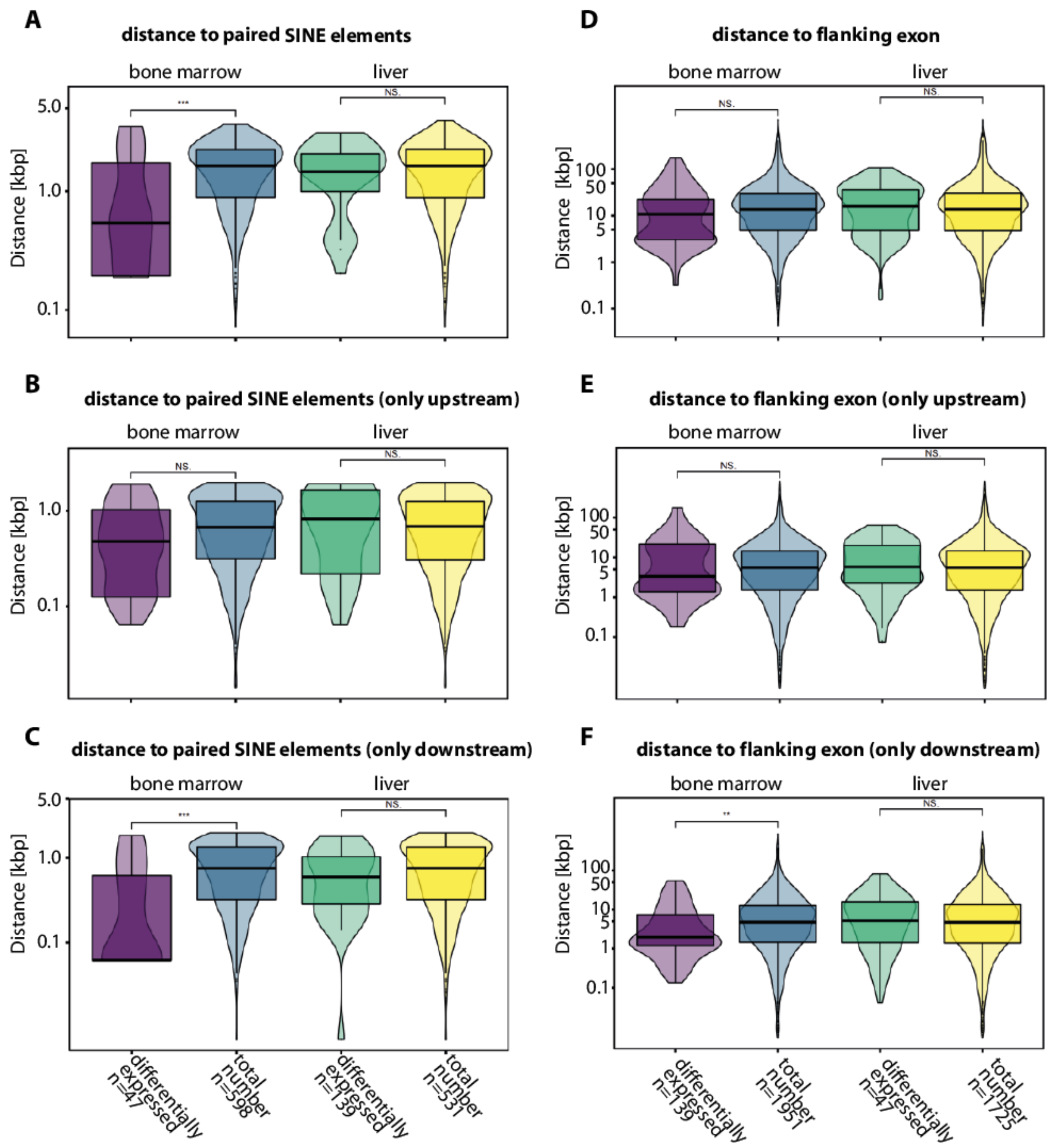

Supplemental Figure 14: The impact of inverted SINE elements and intron length on circRNA expression. (A-F) The distance between circRNAs detectable in bone marrow or liver transcriptomic data to (A-C) the next paired SINE elements or (D-F) the next flanking exon was identified. Subsequently, the distance was plotted for (A-C) all circRNA/SINE elements or (D-F) all circRNA/flanking exons according to circRNA expression status (differentially expressed in *Adar* ko mice or total number). NS: not significant, **: p-value < 0.01, ***: p-value < 0.001.

Supplemental Materials and Methods

Minigene constructs to validate the effect of editing on splicing as predicted by ΔMaxEnt scores

The DNA sequences encompassing the 5’splice site and the surrounding un-edited sequence were ordered from Twist Bioscience (see Supplemental Table 8 for sequences). Subsequently, the DNAs were cloned into the plasmid pcDNA3.1-Gabra3-AdML++ using the restriction enzymes EcoRI and BamHI thereby removing the Gabra3 insert (Licht et al. 2016). Using site-directed mutagenesis pre-edited versions of the plasmids were constructed (Primers for site-directed mutagenesis can be found in Supplemental Table 8). Both plasmid versions were transfected into Hek293 cells using PEI reagent as previously described (Licht et al. 2019). One day after transfection, RNA was isolated using a homemade ‘Trizol’ reagent followed by DNAase I (New England Biolabs, #M0303) digestion according to the manufacturer’s instructions. Reverse transcription was done with 1 µg total RNA using M-MuLV Reverse Transcriptase (New England Biolabs, #M0253S) and random hexamers according to the manufacturer’s instructions. Subsequently, 1/20 of the cDNA was PCR amplified using OneTaq Mix (New England Biolabs, #M0482L) with standard conditions and 28 PCR cycles. PCR reactions were separated on a 1.5% agarose gel, stained with ethidium bromide, and imaged.

Supplemental References

Bajad P, Ebner F, Amman F, Szabo B, Kapoor U, Manjali G, Hildebrandt A, Janisiw MP, Jantsch MF. 2020. An internal deletion of ADAR rescued by MAVS deficiency leads to a minute phenotype. *Nucleic Acids Res* doi:10.1093/nar/gkaa025.

Heraud-Farlow JE, Chalk AM, Linder SE, Li Q, Taylor S, White JM, Pang L, Liddicoat BJ, Gupte A, Li JB et al. 2017. Protein recoding by ADAR1-mediated RNA editing is not essential for normal development and homeostasis. *Genome biology* **18**: 166.

Kerpedjiev P, Hammer S, Hofacker IL. 2015. Forna (force-directed RNA): Simple and effective online RNA secondary structure diagrams. *Bioinformatics* **31**: 3377-3379.

Licht K, Kapoor U, Amman F, Picardi E, Martin D, Bajad P, Jantsch MF. 2019. A high resolution A-to-I editing map in the mouse identifies editing events controlled by pre-mRNA splicing. *Genome research* **29**: 1453-1463.

Licht K, Kapoor U, Mayrhofer E, Jantsch MF. 2016. Adenosine to Inosine editing frequency controlled by splicing efficiency. *Nucleic Acids Res* **44**: 6398-6408.

Lorenz R, Bernhart SH, Honer Zu Siederdissen C, Tafer H, Flamm C, Stadler PF, Hofacker IL. 2011. ViennaRNA Package 2.0. *Algorithms for molecular biology : AMB* **6**: 26.

Vaquero-Garcia J, Barrera A, Gazzara MR, Gonzalez-Vallinas J, Lahens NF, Hogenesch JB, Lynch KW, Barash Y. 2016. A new view of transcriptome complexity and regulation through the lens of local splicing variations. *Elife* **5**: e11752.
